# Complete plastid genome structure of 13 Asian *Justicia* (Acanthaceae) species: comparative genomics and phylogenetic analyses

**DOI:** 10.1186/s12870-023-04532-0

**Published:** 2023-11-15

**Authors:** Zhengyang Niu, Zheli Lin, Yi Tong, Xin Chen, Yunfei Deng

**Affiliations:** 1 Key Laboratory of Plant Resources Conservation and Sustainable Utilization, South China Botanical Garden, Chinese Academy of Sciences, Guangzhou, 510650 China; 2 University of Chinese Academy of Sciences, Beijing, 100049 China; 3 School of Biology and Agriculture, Shaoguan University, Shaoguan, Guangdong 512005 China; 4 School of Chinese Materia Medica Medical, Guangzhou University of Chinese Medicine, Guangzhou, 510006 China; 5 Co-Innovation Center for Sustainable Forestry in Southern China, College of Life Sciences, Nanjing Forestry University, Nanjing, Jiangsu 210037 China

**Keywords:** Asian *Justicia* plants, *Justicia Grossa*, Complete chloroplast genome, Comparative genomics, Phylogeny

## Abstract

**Background:**

*Justicia* L. is the largest genus in Acanthaceae Juss. and widely distributed in tropical and subtropical regions of the world. Previous phylogenetic studies have proposed a general phylogenetic framework for *Justicia* based on several molecular markers. However, their studies were mainly focused on resolution of phylogenetic issues of *Justicia* in Africa, Australia and South America due to limited sampling from Asia. Additionally, although *Justicia* plants are of high medical and ornamental values, little research on its genetics was reported. Therefore, to improve the understanding of its genomic structure and relationships among Asian *Justicia* plants, we sequenced complete chloroplast (cp.) genomes of 12 Asian plants and combined with the previously published cp. genome of *Justicia leptostachya* Hemsl. for further comparative genomics and phylogenetic analyses.

**Results:**

All the cp. genomes exhibit a typical quadripartite structure without genomic rearrangement and gene loss. Their sizes range from 148,374 to 151,739 bp, including a large single copy (LSC, 81,434–83,676 bp), a small single copy (SSC, 16,833–17,507 bp) and two inverted repeats (IR, 24,947–25,549 bp). GC contents range from 38.1 to 38.4%. All the plastomes contain 114 genes, including 80 protein-coding genes, 30 tRNAs and 4 rRNAs. IR variation and repetitive sequences analyses both indicated that *Justicia grossa* C. B. Clarke is different from other *Justicia* species because its lengths of *ndhF* and *ycf1* in IRs are shorter than others and it is richest in SSRs and dispersed repeats. The *ycf1* gene was identified as the candidate DNA barcode for the genus *Justicia*. Our phylogenetic results showed that *Justicia* is a polyphyletic group, which is consistent with previous studies. Among them, *J. grossa* belongs to subtribe Tetramerinae of tribe Justicieae while the other *Justicia* members belong to subtribe Justiciinae. Therefore, based on morphological and molecular evidence, *J. grossa* should be undoubtedly recognized as a new genus. Interestingly, the evolutionary history of *Justicia* was discovered to be congruent with the morphology evolution.

**Conclusion:**

Our study not only elucidates basic features of *Justicia* whole plastomes, but also sheds light on interspecific relationships of Asian *Justicia* plants for the first time.

**Supplementary Information:**

The online version contains supplementary material available at 10.1186/s12870-023-04532-0.

## Introduction

*Justicia* L. is the largest and most taxonomically complex genus in the tribe Justicieae of Acanthaceae [1–8]. It comprises approximately 700 species widely distributed in the tropical and subtropical regions of the world [9, 10]. This genus is characterized by the 2-lipped corolla with the bilobed upper lip and trilobed lower lip, two bithecous stamens, usually one theca above the other and the lower one with a spur at the base [1, 5, 11, 12]. With approximately 150 species, the tropical and subtropical regions of Asia are one of the diversity centers of the genus [3, 5, 7, 13–18]. In Asia, many *Justicia* plants are widely cultivated for ornamental or medical values [3, 19–21]. For example, *Justicia adhatoda* L., *Justicia betonica* L., *Justicia grossa* C.B. Clarke and *Justicia latiflora* Hemsl. are commonly cultivated for ornamental in the gardens [21]. And *J. adhatoda, Justicia gendarussa* N. J. Burman and *Justicia procumbens* L. also have high medicinal value [20, 22, 23]. However, despite high economical values of *Justicia* plants, few reports on its genomics were available [24]. Therefore, to improve our understanding on plastid genomes of these economically important plants and provide useful genetic information for conservation and breeding of them in the future, it is necessary to carry out relevant genetic research.

In addition, due to extensive geographic distributions and high biological diversity, the infrageneric classification of *Justicia* has been controversial for a long time [1, 25–30]. Up to now, there are two divergent approaches in the generic delimitation of *Justicia*. One is to divide *Justicia* into several small segregated genera [25, 26, 31–33], and another is to adopt a broad sense of *Justicia* by dividing into several sections [1, 5, 27–30, 34–39]. In the former, Bremekamp separated *Justicia* s.l. into dozens of genera and published several new genera [26, 31, 32, 40–42]. His treatment was followed by some authors [33, 43–49]. In contrast, Graham [1] adopted a broad concept of the genus and reduced more than 70 names to the synonymies of *Justicia* and divided the genus into nine sections and seven subsections. Her treatment was widely accepted in the recently published flora works [3, 5, 39, 50–55].

However, recent phylogenetic studies indicated that *Justicia* s.l. is a polyphyletic group with its members randomly nested within other genera in tribe Justicieae [9–12], suggesting that previous classification of *Justicia* is problematic. Then, Kiel et al. [9] proposed to divide Justicieae into ten informal clades, of which nine for Old Word (OW) species and one for New Old (NW) species. Although their suggestion on the classification of Justicieae has been most comprehensive until now, molecular data of *Justicia* in their studies were mainly based on samples collected from Africa, Australia and America, but few from Asia. For example, some Asian genera separated from *Justicia*, e.g., *Calymmostachya* Bremek. [32], *Mananthes* Bremek. [26], *Plegmatolemma* Bremek. [32], were not involved in their analysis. Thus, more genetic resources of *Justicia* in Asia need to be supplemented for completion of the evolutionary history of *Justicia* in the future.

The genus *Justicia* from Asia has never been revised except for the regional revisionary works for some countries including China [3, 33], Bangladesh [56], Pakistan [57], Sri Lanka [58], etc. China is one of the diversity centers of *Justicia* in Asia. The most comprehensive works of *Justicia* from China were done by Hu and her colleagues [3, 33]. Hu [33] recognized 44 species in seven genera in Chinse edition of *Flora Reipublicae Popularis Sinica* to follow the narrow sense of generic delimitation proposed by Bremekamp [26, 31, 32]. But, nine years later, Hu et al. [3] adopted the broad sense of generic circumscription proposed by Graham [1] and recorded 43 species in English edition of *Flora of China*. Later, Deng et al. [11] found that *Justicia microdonta* W.W. Sm. is quite different from other *Justicia* plants by having two staminodes and two fertile stamens with both anther-thecae spurred at base, and might be a member of subtribe Graptophyllinae. Therefore, they established a new genus *Wuacanthus* Y.F. Deng et al. for this species. It is implied that the relationships among the remaining Asian species of *Justicia* s. l. are still poorly understood, and thus the further studies on the phylogenetic research among Asian plants is necessary.

The complete cp. genome is characterized by haploid inheritance, relatively small genome and low substitution rates compared with mitochondrial and nuclear genomes, and thus widely used in recent studies of plant phylogeny, phylogeography and population genetics [59–65]. Its molecular structure is highly conservative in most angiosperms, with a double-stranded circular structure divided into four regions, including a large single copy (LSC) region, a small single copy (SSC) region and a pair of inverted repeats (IRs) [66, 67]. It is typically 107–218 kb in genomic size in most land plants, encoding about 100–130 unique genes, mostly containing about 70–80 protein-coding genes, 28–32 tRNAs and 4 rRNAs [68, 69]. Besides, recent advances in sequencing technology and bioinformatic analysis tools have made the acquisition of complete cp. genomes both convenient and cost-effective [70]. Therefore, based on whole cp. genome data, more information sites could be accessible. Thus, our obtained variable sites from whole plastomes are sufficient than previous molecular markers in re-evaluation of the evolutionary histories of some difficult taxa, including some major clades in angiosperms, such as basal lamiid [71] and monocot [72], and other taxa below order, such as Orchidaceae Juss. [63], Ulmaceae Mirb. [73], subtribe Melocanninae Benth. (Poaceae Barnhart) [62], *Horsfieldia* Willd. (Menispermaceae Juss.) [74] and *Oreocnide* Miq. (Urticaceae Juss.) [75]. Additionally, evolutionary rates of coding and non-coding regions of the plastomes are incongruous, suggesting great applicability to screen potential DNA barcodes at various taxonomic levels [76–78]. Therefore, there is no doubt that whole plastid genomes may provide critical insights into historically difficult relationships at different taxonomic levels. Moreover, some IR expansion and contraction events [79], genomic rearrangement [80], gene loss and pseudogenization [81, 82] have also attracted much attention due to their particularities. Thus, the whole cp. genome is definitely an efficient tool for species identification at the genomic level [83–87].

In our study, a total of 12 *Justicia* complete cp. genomes were newly sequenced and assembled, then combined with the previously published cp. genome *J. leptostachya* for further genome comparison analyses. This study aims to (i) understand basic features of *Justicia* plastomes, including genomic size, organization and gene compositions, (ii) find interspecific variation at the genomic structure level, (iii) identify some hypervariable regions and special repetitive sequences for species identification, and (iv) improve our understanding of phylogenetic relationships of these Asian *Justicia* plants, which is also useful to provide baseline information for further completion of the evolutionary history of *Justicia* in the future.

## Results

### Basic characteristics of ***Justicia*** complete chloroplast genomes and nrDNAs

A total of 3,774,489–22,941,320 unique reads were recruited from about 2 Gb clean reads for plastome *de novo* assemblies (Table S1). The average base-coverages of *Justicia* cp. genomes vary from 96X to 521X with 150 bp read length for each sample. The 13 *Justicia* cp. genomes sizes vary from 148,374 bp (*J. latiflora*) to 151,739 bp (*Justicia quadrifaria* (Nees) T. Anderson) and their overall GC content range from 38.1 to 38.4% (Table 1). All the cp. genomes exhibit the identical typical quadripartite structure, comprising of a large single copy region (LSC) from 81,434 bp to 83,676 bp, a small single copy region (SSC) from 16,833 bp to 17,507 bp and a pair of IR regions (IRa/IRb) from 24,947 bp to 25,549 bp.

**Table 1:** General characteristics of 13 Asian Justicia complete chloroplast genomes

	*J. adhatoda*	*J. betonica*	*J. demissa*	*J. gendarussa*	*J. grossa*	*J. latiflora*	*J. leptostachya*
Accession number	MN848249	MN848244	MN885664	MN848252	MN848251	MN848246	MK389502
Total size (bp)	149,503	151,005	150,208	149,735	150,469	148,374	149,227
LSC length (bp)	82,600	82,809	82,326	82,373	82,536	81,434	82,114
SSC length (bp)	17,009	17,182	16,970	17,218	17,507	16,866	16,975
IR length (bp)	24,947	25,507	25,456	25,072	25,213	25,037	25,069
Number of Genes	114	114	114	114	114	114	114
Number of PCGs	80	80	80	80	80	80	80
Number of tRNAs	30	30	30	30	30	30	30
Number of rRNAs	4	4	4	4	4	4	4
Overall GC (%)	38.3	38.3	38.4	38.3	38.4	38.1	38.2
	*** J. lianshanica***	***J. mollissima***	***J. patentiflora***	***J. procumbens***	***J. quadrifaria***	***J. vagabunda***	
Accession number	MN885665	MN848247	MN848248	MN848245	MN848243	MN848250	
Total size (bp)	148,574	150,513	149,018	150,471	151,739	151,247	
LSC length (bp)	81,776	82,811	82,031	82,426	83,676	83,343	
SSC length (bp)	16,868	17,010	16,833	16,947	16,999	17,040	
IR length (bp)	24,965	25,346	25,077	25,549	25,532	25,432	
Number of Genes	114	114	114	114	114	114	
Number of PCGs	80	80	80	80	80	80	
Number of tRNAs	30	30	30	30	30	30	
Number of rRNAs	4	4	4	4	4	4	
Overall GC (%)	38.1	38.2	38.1	38.3	38.2	38.3	

**Fig. 1:**
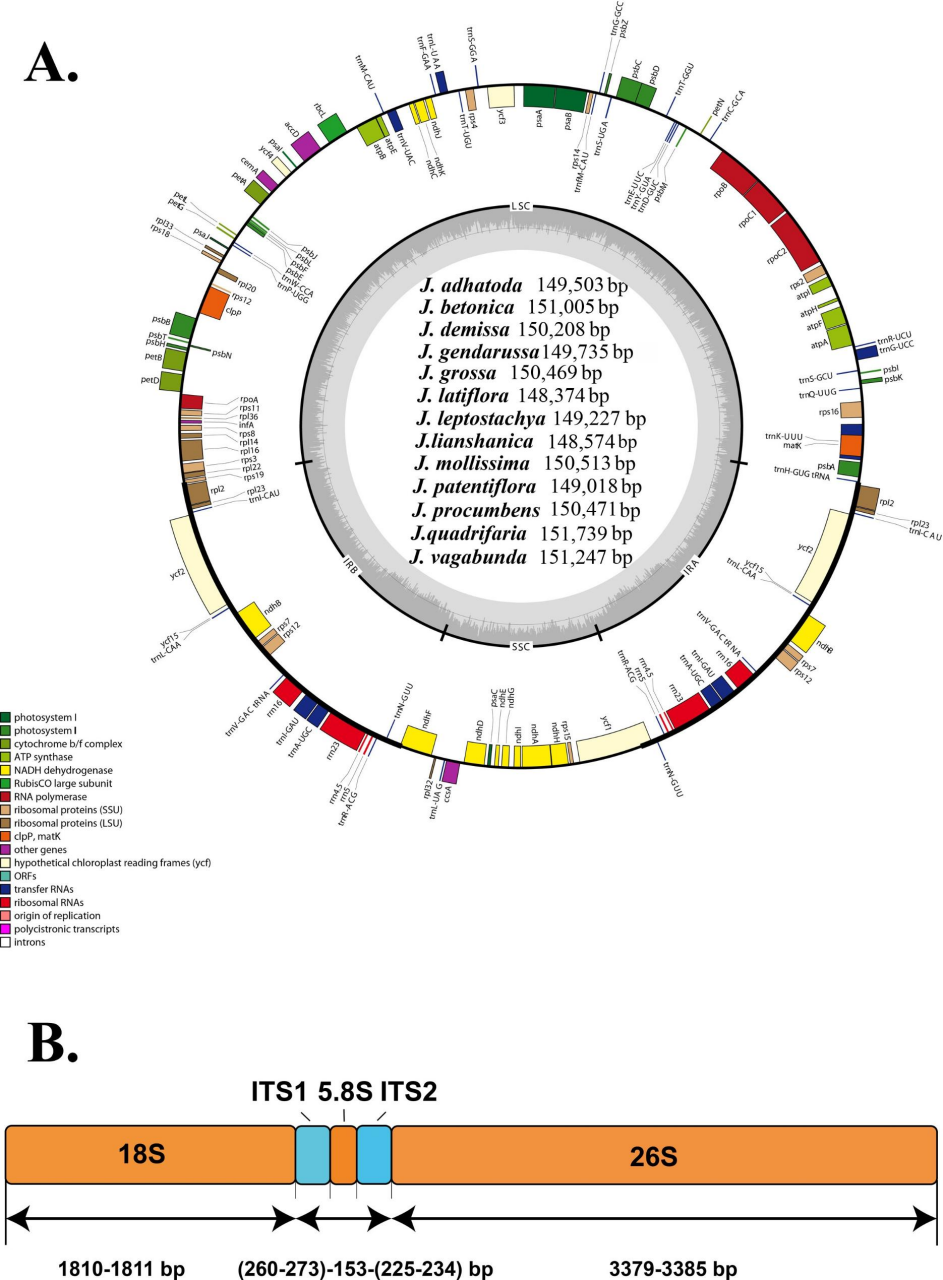
The plastid genome map (**A**) and nrDNA structure (**B**) for the 13 *Justicia* species. The genes drawn on the outside of the circle are transcribed clockwise, while those inside of the circle are transcribed counter clockwise. Genes belonging to different functional groups are color coded. Small single copy (SSC), large single copy (LSC), and inverted repeats (IRa, IRb) are indicated directly

Gene number, order and directions are consistent in the 13 *Justicia* cp. genomes (Fig. 1A). All the cp. genomes share 114 unique genes, containing 80 protein-coding genes, 30 tRNAs and 4 rRNAs (Table 1). According to its location, 62 are located in LSC region, 12 are in SSC region and 6 are in IR regions. As for gene categories, 61 genes are relevant to the gene expression, and 43 genes are associated with photosynthesis (Table 2). According to the sizes of all the protein-coding genes (Table S3), *ycf2* is the longest from 6723 bp (*J. gendarussa*) to 6780 bp (*J. grossa*, *J. demissa* N. H. Xia & Y. F. Deng, *J. mollissima* (Nees) Y. F. Deng & T. F. Daniel and *J. procumbens*), while *petN* is the shortest with 90 bp identical in all the plastomes. Of the 80 unique protein-coding genes, 57 are identical in length among different species, while 23 are variable as such. In addition, 15 genes contain one intron (Table S4), including *atpF*, *ndhA*, *ndhB*, *petB*, *petD*, *rpl2*, *rpl16*, *rpoC1*, *rps12*, *rps16*, *trnA-UGC*, *trnG-UCC*, *trnI-GAU*, *trnK-UUU*, *trnL-UAA*, *trnV-UAC*, *clpP* and *ycf3*, while two genes (*clpP* and *ycf3*) have two introns. Among them, *ndhA*, *rpl2*, *rpoC1*, *rps16* and *ycf3* genes vary in size at the interspecific level.

**Table 2:** Gene contents in the chloroplast genomes of 13 Justicia species

Genes category	Groups of genes	Name of genes
Self-replication	Ribosomal RNAs	*rrn4.5(×2)*, *rrn5(×2)*, *rrn16(×2)*, *rrn23(×2)*
	Transfer RNAs	trnA-UGC*(×2), *trnC-GCA*, *trnD-GUC*, *trnE-UUC*, *trnF-GAA*, *trnfM-CAU*, *trnG-GCC*, trnG-UCC*, *trnH-GUG*, *trnI-CAU(×2)*, trnI-GAU*(×2), trnK-UUU*, *trnL-CAA(×2)*, trnL-UAA*, *trnL-UAG*, *trnM-CAU*, *trnN-GUU(×2)*, *trnP-UGG*, *trnQ-UUG*, *trnR-ACG(×2)*, *trnR-UCU*, *trnS-GCU*, *trnS-GGA*, *trnS-UGA*, *trnT-GGU*, *trnT-UGU*, *trnV-GAC(×2)*, trnV-UAC*, *trnW-CCA*, *trnY-GUA*
	Large ribosmal subunits	rpl2*, *rpl14*, rpl16*, *rpl20*, *rpl22*, *rpl23(×2), rpl32*, *rpl33*, *rpl36*
	Small ribosmal subunits	*rps2*, *rps3*, *rps4*, *rps7(×2)*, *rps8*, *rps11*, rps12**(×2)^*a*^, *rps14*, *rps15*, rps16*, *rps18*, *rps19*
	DNA-dependent RNA polymerase	*rpoA*, *rpoB*, rpoC1*, *rpoC2*
Phytosynthesis	Subunits of photosystem I	*psaA, psaB, psaC, psaI, psaJ*
	Subunits of Photosystem II	*psbA, psbB, psbC, psbD, psbE, psbF, psbH, psbI, psbJ, psbK, psbL, psbM, psbN, psbT, psbZ*
	Subunits of NADH dehydrogenase	ndhA*, ndhB*(×2), *ndhC*, *ndhD*, *ndhE, ndhF*, *ndhG*, *ndhH*, *ndhI*, *ndhJ*, *ndhK*
	Subunits of ATP synthase	*atpA*, *atpB*, *atpE*, atpF*, *atpH*, *atpI*
	Subunits of cytochrome	*petA*, petB*, petD*, *petG*, *petL*, *petN*
	Large subunit of Rubisco	*rbcL*
Other genes	Maturase	*matK*
	Protease	clpP**
	Subunit of acetyl-CoA	*accD*
	Envelope membrane protein	*cemA*
	C-type cytochrome synthesis gene	*ccsA*
	Translation initiation factor	*infA*
Function uncertain	Conserved open reading frames	*ycf1*, *ycf2*, ycf3**, *ycf4*, *ycf15(×2)*

*gene containing one intron, **gene containing two introns, ^*a*^*trans-splinting gene, (×2) shows genes have two copies*

Additionally, some particular genes in *Justicia* plastomes were also identified in our study. First, six genes were determined as partially overlapped genes, including *trnK-UUU*/*matK*, *atpB*/*atpE* and *psbC*/*psbD*. Secondly, *rps12* gene was identified as a trans-splicing gene with 5’ exon located in LSC and 3’ exon duplicated and distributed in two IR regions. Thirdly, the gene *ycf15* in *J. adhatoda* cp genome was found to be about half (63 bp) the length of the others (132 bp).

**Fig. 2:**
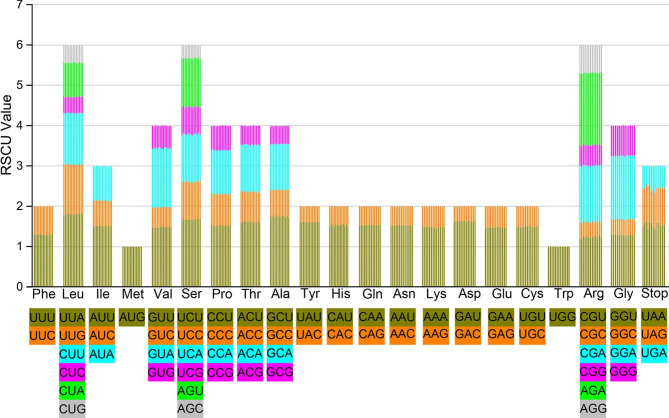
Relative synonymous codon usage (RSCU) in all protein-coding genes of the 13 plastomes. The histogram from the left-hand side of each amino acid shows codon usage value within *Justicia* (From left to right: *J. quadrifaria, J. betonica, J. lianshanica, J. mollissima, J. patentiflora, J. adhatoda, J. vagabunda, J. grossa, J. gendarussa, J. leptostachya, J. procumbens, J. latiflora* and *J. demissa*). Codons encoding 20 amino acids and the stop codon are displayed in rectangular shapes of different colors

According to the codon usage bias analysis, all the protein-coding genes (77,985–78,681 bp) of the 13 *Justicia* cp. genomes, encoding 25,995–26,227 codons, were investigated (Fig. 2, Table S5). Our results showed that all *Justicia* species are similar in amino acid patterns. Among them, Leucine is encoded by the largest number of codons from 2801 to 2852, while Cysteine is the least with 69–79. Besides, a total of 28 codons are directly involved in tRNA synthesis. Most amino acids are encoded with at least two synonymous codons except methionine (Met) and tryptophan (Trp). A total of 77 protein-coding genes identified in *Justicia* cp. genomes started with an AUG codon, but *rps19* and *psbC* start with GUG while *ndhD* contains ACG instead.

For the tandemly repeated nrDNAs, our *de novo* assembly obtained 5,819 bp (*J. grossa*) to 5,846 bp (*J. patentiflora* Hemsl.) comprising 18 S (1,810–1,811 bp), 5.8 S (153 bp), and 26 S (3,379–3,385 bp) ribosomal RNA gene along with two internal transcribed spacer (ITS) I (260–273 bp) and II (225–234 bp) in the middle (Fig. 1B).

### IR contraction and expansion

**Fig. 3:**
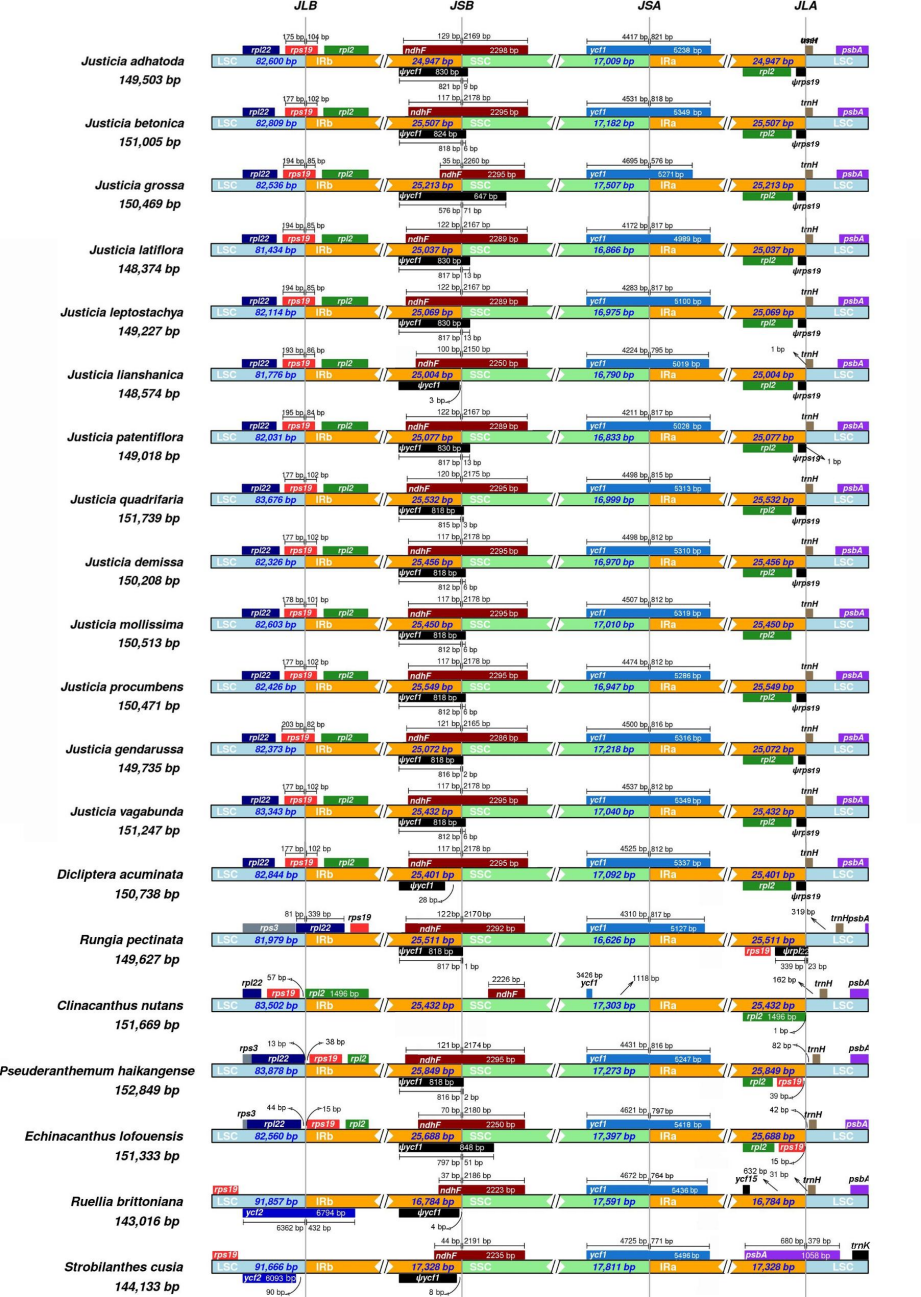
Comparison of IR/SC boundary regions of the 13 *Justicia* species and seven species of other genera of Acanthaceae

In our study, IR/SC junctions of cp. genomes of the 13 *Justicia* species and seven species of other genera in Acanthaceae were compared and visualized (Fig. 3). First, the gene *rps19* stretches across LSC and IRb regions of cp. genomes of all *Justicia* species and *Dicliptera acuminata* (Ruiz & Pav.) Juss., with 5′ end of the *rps19* located in the IRbs (82–104 bp) and 3′ end located in the LSCs (175–203 bp). Therefore, *rps19* gene creates a pseudogene of the 5′ end of this gene (*Ψrps19*) in IRa. However, in *Rungia pectinata* (L.) Nees and *Ruellia brittoniana* Leonard, it is found that *rpl22* and *ycf2* gene rather than *rps19* gene span the junction of LSC/IRb borders. Meanwhile, the gene *rpl22* duplicates a pseudogene (*Ψrpl22*) in the border of LSC/IRa of *R. pectinata*, but *ycf2* gene in *R. brittoniana* not as such. Different from others, genes *rpl22*, *rps19* and *ycf2* are closed to the junction of LSC/IRb in plastomes of *Clinacanthus nutans* (Burm.f.) Lindau, *Pseuderanthemum haikangense* C. Y. Wu et H. S. Lo, *Echinacanthus lofuensis* (H.Lév.) J.R.I.Wood and *Strobilanthes cusia* (Nees) Kuntze. Secondly, the tRNA genes *trnH-GUG* and *Ψrps19* are adjacent to the junctions of LSC/IRa in cp. genomes of *Justicia* and *D. acuminata*. However, the genes *rps19* in *P. haikangense* and *E. lofuensis* are duplicated due to this gene fully located in IRs. Additionally, *rpl2* and *ycf15* gene are adjacent to the LSC/IRa borders of *C. nutans* and *R. brittoniana* while (*Ψ*) *rps19* genes are adjacent to the same locations in others. Particularly, *psbA* was found to be a crossing gene within the LSC/IRa border of *S. cusia*. Thirdly, it is discovered that *ycf1* genes of plastomes of most genera span SSC/IRa border with the exception of *C. nutans* fully located in SSC region with 1,118 bp far away from the junction. Notably, the *ycf1* gene is only 576 bp in IRa region of *J. grossa* cp. genome, but about 800 bp in all of the other species. Fourthly, the pseudogene *Ψycf1* is a part of *ycf1* protein-coding gene copy with the 5′ end located in the IRb region, with the sizes of 647 bp (*J. grossa*) to 848 bp (*E. lofouensis*). Meanwhile, most *Ψycf1* can cross SSC and IRb regions, but those of *Justicia lianshanica* (H.S.Lo) H.S.Lo, *D. acuminata*, *R. brittoniana* and *S. cusia* are fully located in IRb regions. Most *ndhF* genes are within the SSC/IRb borders with the exception of *C. nutans* fully located in SSC region. Notably, the length of *ndhF* gene in IRb of *J. grossa* plastome is shorter (35 bp) than that in other *Justicia* species (100–129 bp), but similar in length with *R. brittoniana* (37 bp) and *S. cusia* (44 bp).

### Genome divergence comparison

**Fig. 4:**
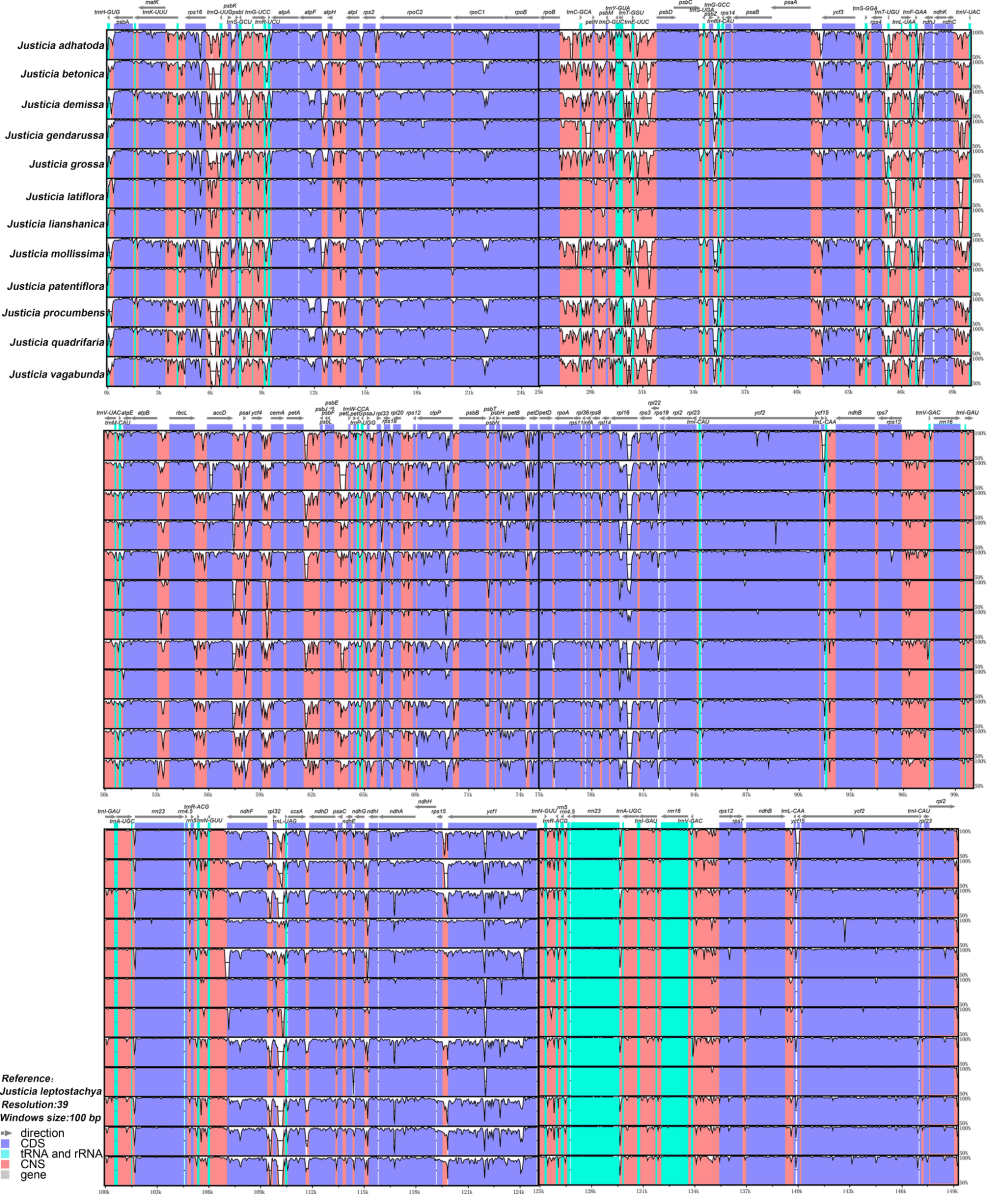
Genome divergence plots of the 13 cp. genomes with *J. leptostachya* as a reference based on visualized global alignment. Grey arrows and thick black lines above the alignment indicate genes with their orientations and directions. Protein-coding regions (exon), non-coding regions (CNS) and untranslated regions (UTR) are marked in red, blue and green, respectively. A cut-off of 70% identity was used for the plots, and the Y-scale represents the percent identity from 50–100%

To visualize hypervariable regions, multiple sequence alignments were implemented using the program mVISTA (Fig. 4). The divergence of non-coding regions (CNS) is greater than that of coding regions (CDS), while LSC and SSC regions are more variable than IR regions. According to global alignment, the most highly divergent regions in intergenic spacer are *rps16-trnQ*, *trnS-trnG*, *atpF-atpH*, *rpoB-trnC*, *trnE-psbD*, *psbZ-trnfM*, *rps4-trnT*, *trnF-ndhJ*, *ndhC-trnV*, *petA-psbJ*, *psbE-petL*, *rpl32-trnL* and *rps15-ycf1*, while divergent regions in coding regions are *atpF*, *rpl16* and *ycf1*.

**Fig. 5:**
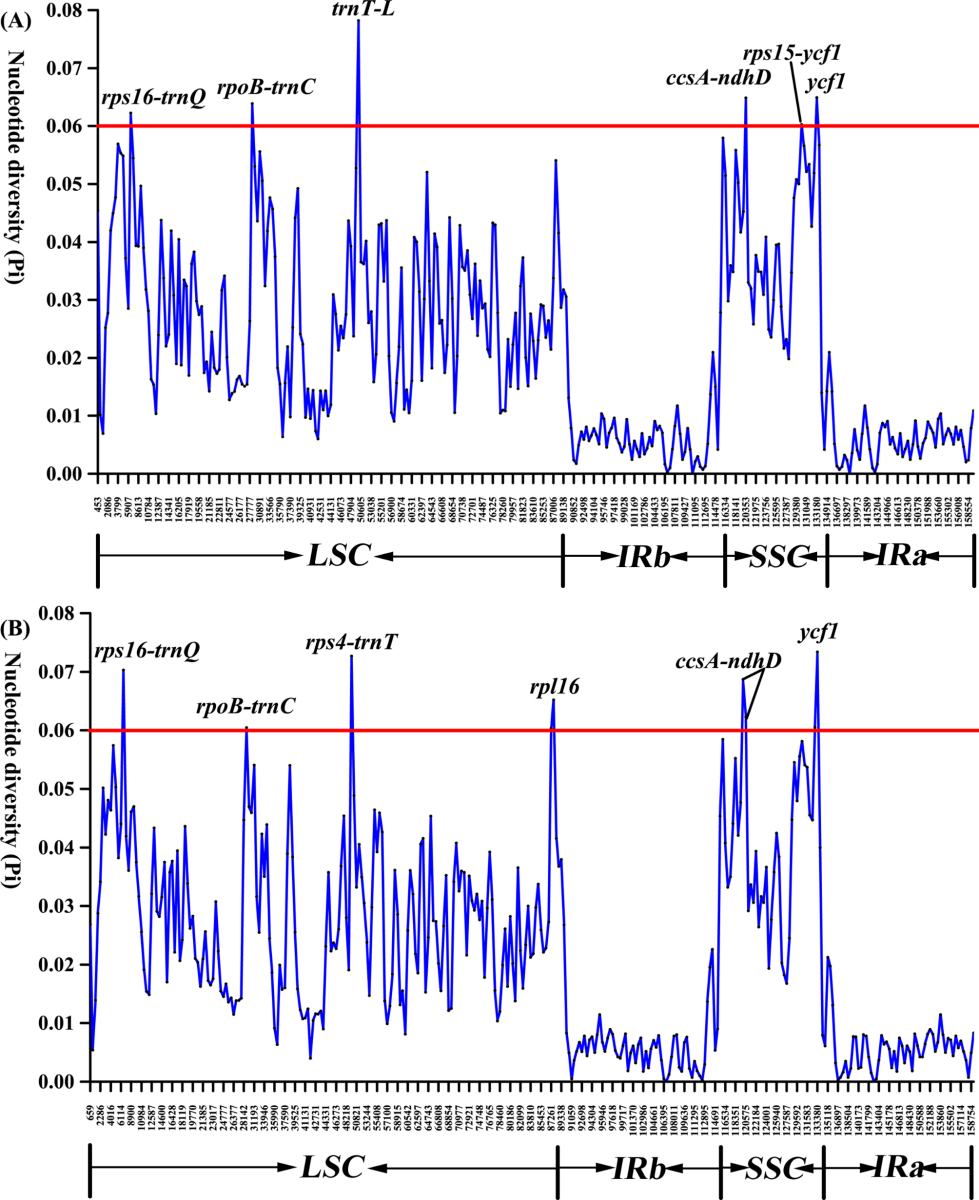
Sliding window analysis of the 13 cp. genomes alignment. Window length: 600 bp; step size: 200 bp. X-axis: position of the midpoint of a window. Y-axis: nucleotide diversity of each window. (**A**) Nucleotide diversity of A-clade dataset; (**B**) Nucleotide diversity of B-clade dataset

A sliding window was used to compare hotspots regions among 13 *Justicia* species. 693 representative loci were divided into two groups, which are composed of two clades of staggered loci (Fig. 5A and B). The nucleotide diversity (Pi) value enormously ranges from 0 to 0.072, and the mean value is 0.0219. In general, Pi value of SC regions is significantly greater than that of IR regions. To exactly analyze interspecific variations, eight highly variable regions (Pi > 0.06) were identified, including *trnT-trnL* (Pi = 0.07944), *ycf1* (Pi = 0.07521), *rps4-trnT* (Pi = 0.07203), *rps16-trnQ* (Pi = 0.06823), *ccsA-ndhD* (Pi = 0.06671), *rpoB-trnC* (Pi = 0.06387), *rpl16* (Pi = 0.06316), and *rps15-ycf1* (Pi = 0.06047). According to their locations, six of them are located in LSC region while two are in SSC.

### Repetitive sequences analyses

**Fig. 6:**
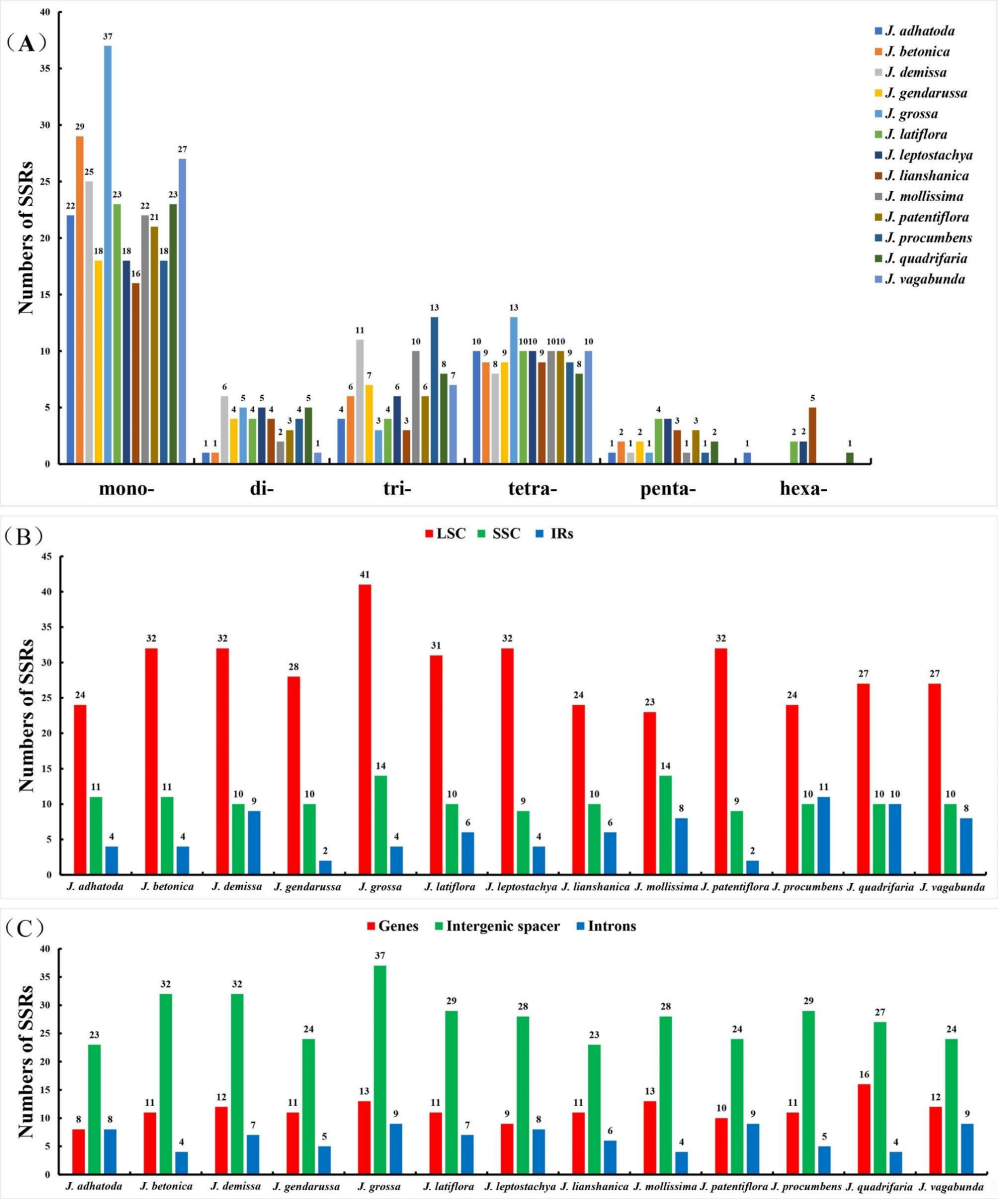
Distribution of SSRs in the chloroplast genomes of 13 *Justicia* species. (**A**) Number of different SSRs types; (**B**) SSRs distribution in LSC, SSC and IR regions; (**C**) SSRs distribution between genes, intergenic spacer and introns

As short tandem repeats with 1–6 nucleotides units, SSRs are widely dispersed in *Justicia* plastomes. The number of SSRs varies unevenly in 13 *Justicia* cp. genomes (Fig. 6, Table S6). Statistically, SSRs fluctuate within a range of 39–59, comprising 23–41 SSRs in LSC regions, 9–14 SSRs in SSC regions and 2–11 SSRs in IR regions (Fig. 6B). According to genomic regions, 8–16 SSRs were identified in coding genes, 23–37 SSRs in intergenic spacer and 4–9 SSRs in introns (Fig. 6C). Most SSRs were detected in LSC and intergenic spacer regions, whereas few SSRs were detected in IR regions and introns. The most abundant SSRs (59) were identified in *J. grossa*, while the others range from 39 (*J. adhatoda*) to 51 (*J. demissa*). For base contents of SSRs, all the *Justicia* cp. genomes are made up of 90% A/T and 10% C/G bases (Table S6). Among them, mononucleotide is the most abundant units and hexanucleotide was only identified in five species including *J. leptostachya*, *J. latiflora*, *J. quadrifaria*, *J. adhatoda* and *J. lianshanica*, of which *J. lianshanica* has the largest number (5) (Table 3; Fig. 6A). Notably, *J. grossa* has the largest number of mononucleotide (37) and tetranucleotide (13) repeats compared with other members of *Justicia* (Fig. 6A).

**Table 3:** The polymorphic SSRs among 13 Justicia chloroplast genomes

Type	*J. adhatoda*/*J. betonica*/*J. demissa*/*J. gendarussa*/ *J. grossa*/*J. latiflora*/*J. leptostachya*/*J. lianshanica*/ *J. mollissima*/*J. patentiflora*/*J. procumbens*/ *J. quadrifaria/J. vagabunda*	Location	Regions
ATT	0/0/0/0/0/0/0/0/4/0/0/0/0	*trnH-GUG-psbA*	LSC
TTC	0/0/0/4/0/0/0/0/0/0/0/0/0	*trnH-GUG-psbA*	LSC
ATTAA	0/0/0/0/0/3/3/3/0/0/0/0/0	*trnK-UUU-rps16*	LSC
TATT	0/3/0/0/0/0/0/0/0/0/0/0/0	*trnK-UUU-rps16*	LSC
T	0/0/0/0/0/0/0/0/0/0/0/0/0/11	*trnK-UUU-rps16*	LSC
C	0/0/10/10/0/0/0/0/11/0/0/0/0	*trnK-UUU-rps16*	LSC
TTGAAA	3/0/0/0/0/0/0/0/0/0/0/0/0	*rps16* intron	LSC
ATTG	3/0/3/3/3/0/0/0/3/0/3/3/3	*rps16* intron	LSC
TTTC	0/0/0/0/0/0/0/0/0/0/0/0/3	*rps16* intron	LSC
A	0/0/0/12/14/11/0/11/10/0/0/0/0	*rps16-trnQ-UUG*	LSC
AAT	0/0/0/0/0/0/0/0/0/0/5/0/0	*psbK-psbI*	LSC
AT	0/0/8/6/6/7/7/7/0/9/7/0/0	*psbK-psbI*	LSC
T	13/10/10/10/0/10/0/0/0/0/0/0/0	*psbK-psbI*	LSC
A	0/0/10/10/13/0/0/0/0/0/0/16/0	*psbI-trnS-GCU*	LSC
T	11/12/0/0/0/10/10/0/0/0/0/15/0	*trnR-UCU-atpA*	LSC
A	12/14/0/10/12/10/13/11/0/12/0/0/0	*atpF* intron	LSC
T	11/10/0/0/10/0/0/0/0/0/0/0/0	*atpF-atpH*	LSC
A	0/0/0/0/10/0/0/0/0/0/0/0/0	*atpF-atpH*	LSC
A	0/16/0/0/0/0/0/0/0/0/0/12/15	*atpH-atpI*	LSC
A	12/0/11/12/12/16/13/10/13/11/11/11	*rps2-rpoC2*	LSC
AATTCA	0/0/0/0/0/0/0/0/0/0/0/3/0	*rpoC2*	LSC
T	0/0/0/0/0/0/0/0/0/10/0/0/0	*rpoC2*	LSC
C	0/13/0/0/10/12/17/13/0/19/0/0/0	*rpoC1* intron	LSC
ATT	0/0/0/0/0/0/0/0/0/0/0/4/0	*rpoB-trnC-GCA*	LSC
TATTAA	0/0/0/0/0/0/0/3/0/0/0/0/0	*trnC-GCA-petN*	LSC
A	0/0/0/0/0/10/0/0/0/12/0/0/0	*trnC-GCA-petN*	LSC
ATAG	0/3/0/3/3/0/0/0/0/0/0/0/0	*petN-psbM*	LSC
AT	0/0/6/0/0/0/0/0/0/0/6/0/0	*petN-psbM*	LSC
ATTT	3/0/0/0/0/0/0/0/0/0/0/0/3	*psbM-trnD-GUC*	LSC
TTTA	0/0/0/0/0/3/3/3/0/3/0/0/0	*psbM-trnD-GUC*	LSC
CAATA	3/3/0/0/3/3/3/0/0/3/0/3/0	*trnE-UCC-trnT-GGU*	LSC
T	10/17/0/0/10/0/0/0/0/0/0/0/10	*trnE-UCC-trnT-GGU*	LSC
T	11/0/10/10/0/11/10/0/0/10/13/11/0	*trnT-GGU-psbD*	LSC
ATTA	0/0/0/0/0/3/3/0/0/3/0/0/0	*trnT-GGU-psbD*	LSC
A	11/0/0/0/0/13/10/13/0/10/0/0/0	*psbZ-trnG-GCC*	LSC
T	0/0/0/0/11/0/0/0/0/0/0/0/0	*psbZ-trnG-GCC*	LSC
AT	0/0/0/0/0/0/0/0/8/0/0/0/0	*ycf3-trnS-GGA*	LSC
A	0/0/0/0/10/0/0/0/0/0/0/0/0	*ycf3-trnS-GGA*	LSC
TTA	0/4/0/0/0/0/0/0/0/0/0/0/0	*trnS-GGA-rps4*	LSC
TTTC	0/3/0/0/0/0/0/0/0/0/0/0/0	*rps4-trnT-UGU*	LSC
ATAG	0/0/0/4/0/0/0/0/0/0/0/0/0	*rps4-trnT-UGU*	LSC
AT	0/0/0/0/0/6/7/6/0/0/0/7/0	*rps4-trnT-UGU*	LSC
TTTC	0/0/0/0/0/3/3/3/0/3/0/0/0	*trnT-UGU-trnL-UAA*	LSC
AT	6/0/6/0/6/7/6/7/6/6/6/6/0	*trnT-UGU-trnL-UAA*	LSC
TA	0/0/0/6/0/0/0/0/0/0/8/0/7	*trnT-UGU-trnL-UAA*	LSC
GTTG	0/0/0/0/0/3/3/3/0/3/0/0/0	*trnF-GAA-ndhJ*	LSC
ATT	0/0/0/4/0/0/0/0/0/0/0/0/0	*trnF-GAA-ndhJ*	LSC
T	0/0/12/0/15/0/0/0/0/0/0/0/0	*trnF-GAA-ndhJ*	LSC
TTA	0/0/0/0/0/0/0/0/4/0/0/0/0	*ndhC-trnV-UAC*	LSC
T	14/10/10/10/17/10/10/0/0/14/0/10/10	*ndhC-trnV-UAC*	LSC
ATA	0/0/4/4/5/4/4/0/0/4/4/0/0	*atpB-rbcL*	LSC
T	0/0/0/0/11/0/0/0/0/16/16/10	*atpB-rbcL*	LSC
A	0/0/0/0/12/0/0/11/0/0/0/0/0	*rbcL-accD*	LSC
ATTA	0/0/0/0/0/0/0/0/0/0/4/4/0	*accD-psaI*	LSC
TTAA	0/0/0/0/0/0/3/3/0/3/0/0/0	*accD-psaI*	LSC
T	0/12/11/11/0/10/0/0/12/0/12/12/11	*psaI-ycf4*	LSC
GAAA	0/0/0/0/3/0/0/0/0/0/0/0/0	*ycf4-cemA*	LSC
ATA	0/0/4/0/0/0/0/0/4/0/4/4/0	*psbE-petL*	LSC
A	0/0/0/0/0/0/0/0/0/0/0/0/11	*psbE-petL*	LSC
T	0/0/0/0/0/0/0/0/0/0/0/16/0	*psaJ-rpl33*	LSC
T	0/0/0/12/14/19/10/12/0/12/0/0/0	*rpl20-rps12*	LSC
TTTC	0/0/0/3/3/3/3/3/0/3/0/0/0	*clpP* intron	LSC
AT	0/0/0/0/0/6/8/6/0/6/0/0/0	*clpP* intron	LSC
T	0/0/10/0/0/0/0/0/0/0/0/0/12	*clpP* intron	LSC
T	10/0/12/0/11/0/10/0/0/15/11/0/0	*petB* intron	LSC
TTTA	0/0/0/0/3/0/0/0/0/0/0/0/0	*petB-petD*	LSC
T	0/0/0/0/16/0/0/0/0/0/0/0/10	*rpoA*	LSC
AAAT	3/0/0/0/0/0/0/0/0/0/0/0/0	*rpl16* intron	LSC
TTTC	0/3/0/0/0/0/0/0/0/0/0/0/0	*rpl16* intron	LSC
AATA	0/0/0/3/0/0/0/0/0/0/0/0/0	*rpl16* intron	LSC
TTA	0/0/0/0/0/0/0/0/0/0/0/0/4	*rpl16* intron	LSC
T	0/10/0/0/12/0/0/0/0/0/0/0/10	*rpl16* intron	LSC
AATAAG	0/0/0/0/0/0/0/3/0/0/0/0/0	*rps12-trnV-GAC*	IR
T	0/0/0/0/10/0/0/0/0/0/0/0/0	*rps12-trnV-GAC*	IR
TTTAA	0/0/0/3/0/3/3/3/0/3/0/0/0	*trnR-ACG-trnN-GUU*	IR
ATT	4/0/0/0/0/0/0/0/0/0/0/0/0	*trnR-ACG-trnN-GUU*	IR
T	13/18/15/0/10/0/0/0/16/0/15/11/11	*trnR-ACG-trnN-GUU*	IR
T	0/10/0/0/15/0/0/0/0/0/0/0/0	*ndhF*	SSC
T	0/0/0/0/11/0/0/0/0/0/0/0/0	*rpl32-trnL-UAG*	SSC
AATA	3/3/3/3/0/3/3/3/3/3/3/3/3	*ndhD*	SSC
A	10/10/0/0/10/10/11/0/10/0/0/12	*ndhD-psaC*	SSC
A	0/0/0/10/0/0/14/0/11/0/0/0/0	*ndhG*	SSC
AATC	3/3/3/0/3/0/0/0/3/0/3/0/3	*rps15-ycf1*	SSC
TTTG	0/0/0/0/0/0/0/0/3/0/0/3/0	*rps15-ycf1*	SSC
T	0/14/0/0/0/0/0/0/0/0/0/0/0	*rps15-ycf1*	SSC
AATT	3/0/3/3/3/3/3/3/3/3/3/3/3	*ycf1*	SSC
TTTC	0/0/0/0/3/0/0/0/0/0/0/0/0	*ycf1*	SSC
TTA	0/0/0/0/0/4/4/4/0/4/0/0/0	*ycf1*	SSC
TCT	8/5/6/4/10/6/5/6/6/6/5/6/6	*ycf1*	SSC
T	13/13/16/12/16/12/12/12/13/12/16/16/13	*ycf1*	SSC

**Fig. 7:**
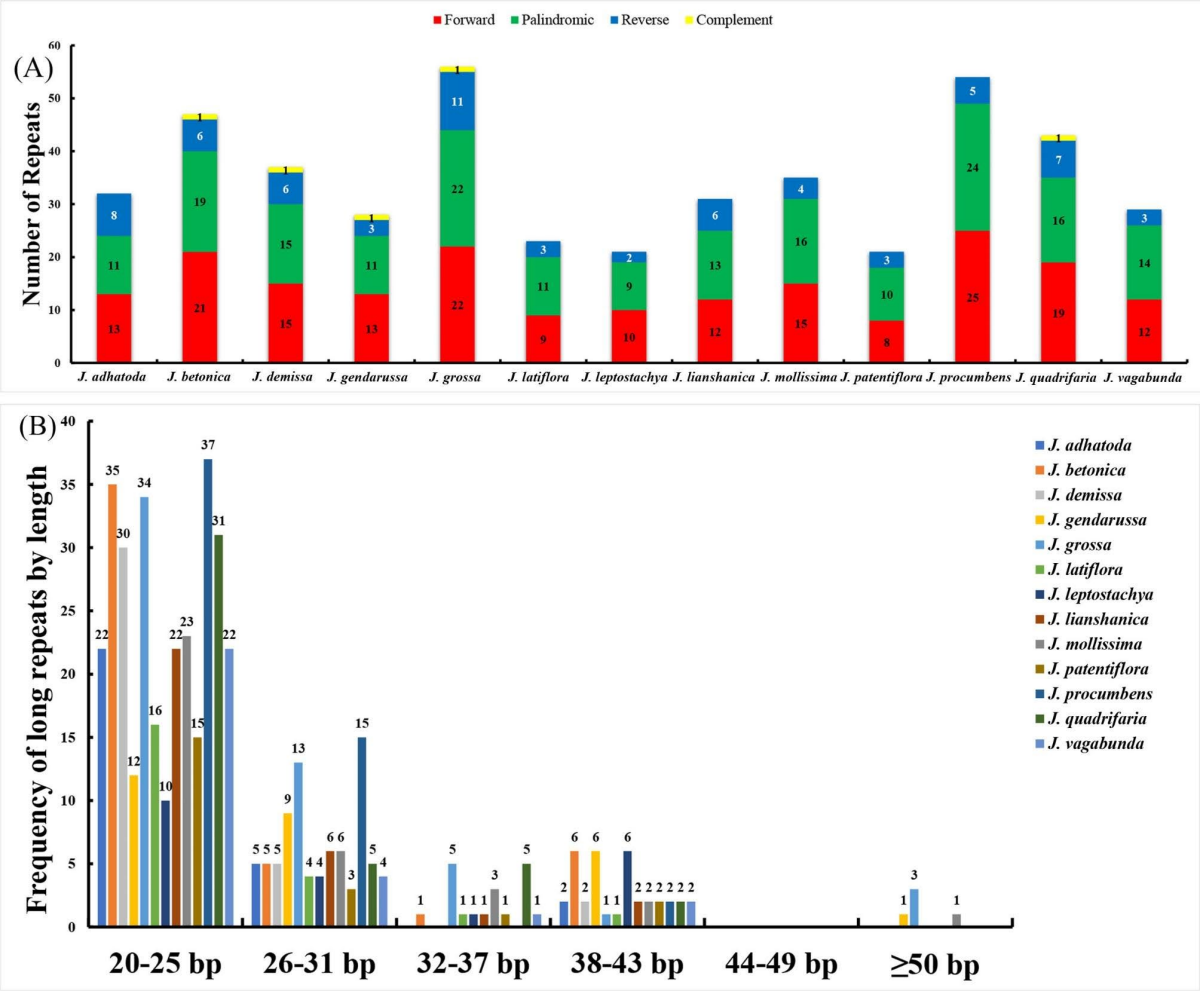
Dispersed repeats of the 13 *Justicia* cp. genomes. (**A**) Number of four repeat types; (**B**) Frequency of long repeats by length

A total of 22–57 dispersed repeats were also defined in the 13 cp. genomes, including forward, palindromic, reverse and complement repeats (Fig. 7A). Among them, palindromic repeats are the richest in all the *Justicia* cp. genomes. Besides, the maximum number of dispersed repeats were detected in *J. grossa* (56) compared with others. In terms of repeat length, most dispersed repeats concentrate on lengths of 20–25 bp, with the exception of three species having dispersed repeats of over 50 bp, including *J. gendarussa* (1), *J. mollissima* (1) and *J. grossa* (3) (Fig. 7B).

### Phylogenetic analysis

The whole cp. genome data matrix consisting of 62 sequences is 188,699 bp in total length. It is characterized by sequence divergence with 56,714 variable sites, including 38,522 parsimony informative sites and 18,192 singleton variable sites. The ITS data matrix made up of 63 sequences is 988 bp in total length. It is characterized by sequence divergence with 457 variable sites, including 312 parsimony informative sites and 145 singleton variable sites.

**Fig. 8:**
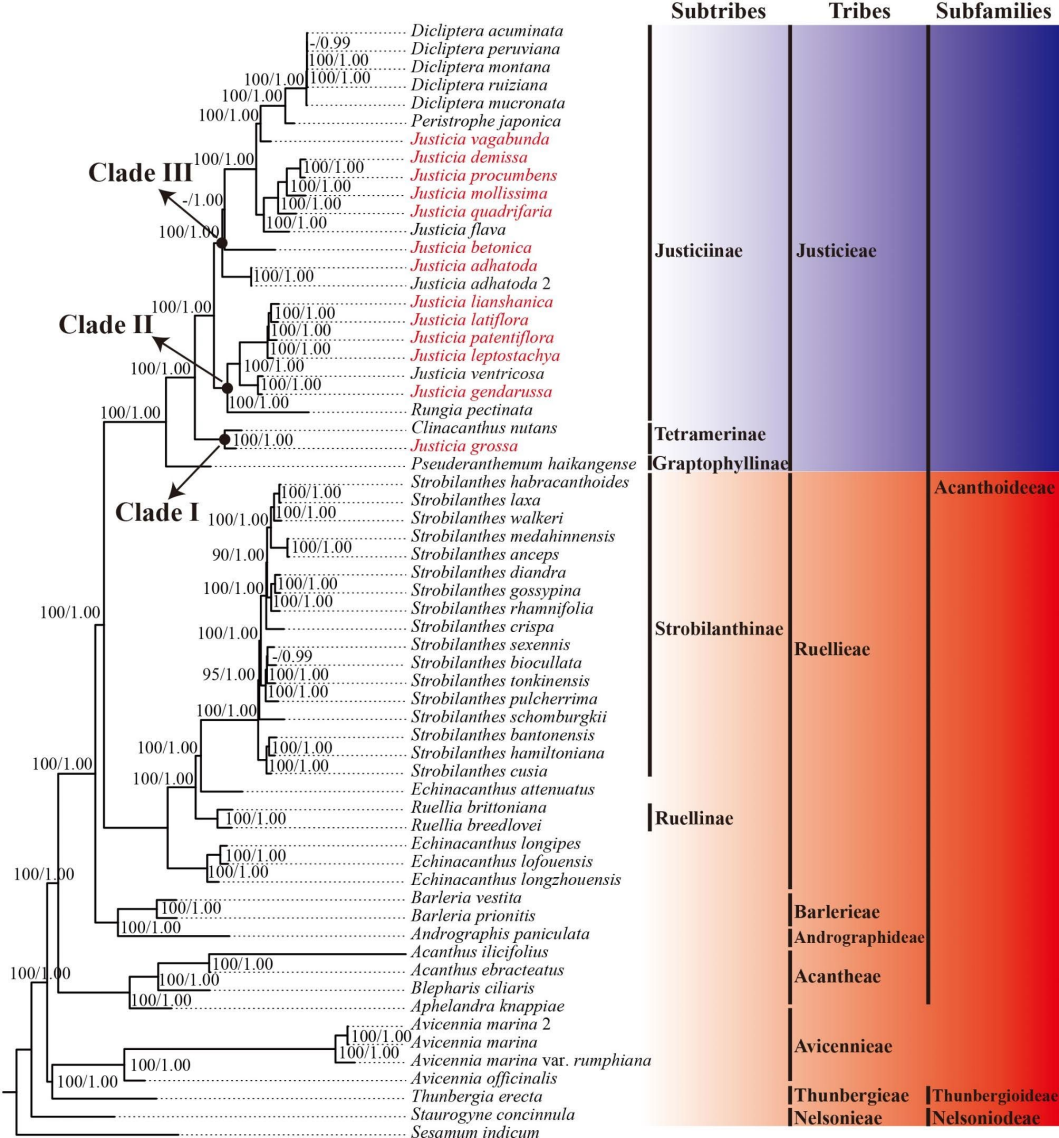
Phylogenetic tree reconstruction for *Justicia* species and other genera of Acanthaceae based on whole chloroplast genome (WCG) by using Maximum likelihood (ML) and Bayesian inference (BI) methods. Different Asian sections of *Justicia* are marked with different colors. The tribe Justicieae are printed in blue while the other tribes of Acanthaceae are printed in red. Only bootstrap values (BS) ≥ 70% and posterior probabilities (PP) ≥ 0.95 are indicated at each node

Because the reconstructed ML tree and BI tree share the same topology, we only showed the ML phylogram with bootstrap (BS) and posterior probability (PP) values labeled near each node (Fig. 8). Our phylogenetic results indicated that phylogenetic relationships of the 13 *Justicia* species based on three datasets (WCG, PCG and ITS) exhibit identical tree topologies (Fig. 8, Fig. S1–S4).

According to our phylogenetic results, a robust phylogenetic framework for four subfamilies of Acanthaceae is as follows: (Nelsonioideae(Acanthoideae(Thunbergioideae + Avicennioideae))). Additionally, the stable framework of most tribes of Acanthaceae with the exception of Neuracantheae and Whitfieldieae is also exhibited, that is (Nelsonieae((Acanthaceae((Andrographideae + Barlerieae) (Justicieae + Ruellieae))) (Thunbergieae + Avicennicae))) (Fig. 8). Importantly, all the nodes of subfamilies and tribes are strongly supported (BS = 100, PP = 1.0) in our study.

Additionally, our results also strongly support (BS = 100, PP = 1.0) that *Justicia* is a polyphyletic group and suggest to divide all sampled *Justicia* species in the present study into three informal clades—Clade I, II and III (Fig. 8). In Clade I, *J. grossa* (the type of *Justicia* sect. *Grossa*) is the earliest diverging species sister to the monospecific genus *Clinacanthus*. And both of them belong to subtribe Tetramerinae of tribe Justicieae. However, Clade II and III consist of all the remaining *Justicia* species and three other genera, which belong to subtribe Justiciinae of tribe Justicieae. Clade II includes a single sampled species of *Rungia* Nees and six sampled species of *Justicia*, including *J. gendarussa*, *J. ventricosa*, *J. lianshanica, J. latiflora, J. patentiflora* and *J. leptostachya*. Within this clade, *Rungia* is the earliest diverging genus. Then, *J*. *gendarussa* and *J. ventricosa* form a sister subclade with the remaining four species (BS = 100, PP = 1.0). Clade III is sister to Clade II with strong support values (BS = 100, PP = 1.0). This clade contains *Peristrophe japonica* (Thunb.) Bremek., five sampled species of *Dicliptera* Juss. and nine sampled species of *Justicia*. Within Clade III, *J. adhatoda* and *J. betonica* are prior diverging species and form two separate subclades. Then, the African species *J. flava* forms a subclade with four other Asian *Justicia* species, including *J. quadrifaria*, *J. demissa, J. procumbens* and *J. mollissima* (BS = 100, PP = 1.0). However, *Justicia vagabunda* Benoist is distantly related to other members of *Justicia* but sister to *Dicliptera* and *Peristrophe* Nees with strong support values (BS = 100, PP = 1.0).

## Discussion

### Basic features and genomic variation of ***Justicia*** cp. genomes

The complete cp. genome often tracks back maternal line inheritance in contrast to the nuclear genome [88]. Therefore, due to its highly conserved structure, limited sequence length and countable genes, it is widely used in recent studies of genetic variation, genomic evolution and phylogeny [59–65, 87–90]. In our study, the 13 *Justicia* cp. genomes exhibit a typical quadripartite structure, with two distinct single-copy regions separated by two inverted repeat (IR) regions (Fig. 1). All the cp. genomes are similar in genomic structure, gene composition and order (Tables 1 and 2; Fig. 3), which is consistent with other genera in Acanthaceae [91, 92]. Despite the overall conservation in structure, *Justicia* whole plastomes vary from 148,374 bp (*J. latiflora*) to 151,739 bp (*J. quadrifaria*) in size, falling within the middle range (120–218 kb) of land plants [66]. Compared with those of previously reported genera in Acanthaceae, *Justicia* plastid genomes are generally smaller than *Barleria* L. (151,977–152,324 bp) [93, 94], *Echinacanthus* Nees (152,384–152,672 bp) [91], *Aphelandra knappiae* Wassh. (152,457 bp) [95], *P. haikangense* (152,849 bp) [96] and *Thunbergia erecta* Nees (152,202 bp) [97], but larger than *Strobilanthes* Blume (144,012–145,110 bp) [98–100]. Additionally, some *Justicia* plants also have similar genome sizes with its closely related genera (Fig. 8). For example, the cp. genome size of *J. gendarussa* (149,735 bp) is close to that of *R. pectinata* (149,627 bp) [101], and the genome sizes of three species belonging to *Justicia* sect. *Rostellaria* (*J. demissa*, *J. mollissima* and *J. procumbens*) (~ 150 kb) are similar to that of *Dicliptera* [92]. It is indicated that the length of the cp. genome sequence is quite variable among different species within *Justicia*. Additionally, the LSC length extends quite large from 81,434 bp (*J. latiflora*) to 83,676 bp (*J. quadrifaria*), however, the IR length is less variable between 24,947 bp (*J. adhatoda*) to 25,549 bp (*J. procumbens*). The most considerable length of SSC region was recorded in *J. grossa* (17,507 bp), while the others are between 16,790 bp (*J. lianshanica*) to 17,218 bp (*J. gendarussa*) (Table 2). It is implied that greater sequence length divergence was observed in LSC and SSC, while fewer sequence differences were found in the two IR regions.

All the cp sequences consist of 114 unique genes, which is same as those in other genera of Acanthaceae, including *Aphelandra* R.Br., *Dicliptera* and *Rungia* [92, 95, 101], but fewer than those of *Barleria* (131) [94]. The codon usage bias of 20 amino acids among different species is similar (Fig. 2, Table S5), which is congruent with other angiosperms [65, 89]. It is revealed that most protein-coding genes are generally identical, but genes *accD*, *matK*, *ndhI*, *rpl22*, *rpl20*, *rpoA*, *rps16*, *rps18*, *ycf1* and *ycf2* of *J. grossa* are obviously different from those of other *Justicia* plants in length and base variation (Table S3), suggesting *J. grossa* is different from other *Justicia* plants in plastid genes. Regarding the initiation codon of *ndhD*, ACG is commonly used as an alternative to AUG in many land plants, but it can still be converted to a functional AUG initiator codon by RNA editing [102–104], probably depending on a molecular cofactor PPR protein CRR4 during transcription [105]. Additionally, GUG is also reported as an initiation codon of *psbC* and *rps19* in other plants, such as *Thalictrum* L. (Ranunculaceae Juss.) [104], *Garcinia* L. (Clusiaceae Lindl.) [106], *Populus* L. (Salicaceae Mirb.) [107] and *Betula platyphylla* Suk. (Betulaceae Gray) [108], but these two genes cannot be edited back to AUG. However, recent studies suggested that an important translation initiation signal, known as Shine-Dalgarno (SD) sequence, can interact with 3’ end of the 16 S rRNA and facilitate translation initiation from the GUG [108, 109], which is responsible for expression of *psbC* and *rps19* in chloroplast. The *ycf15* gene is often duplicated in IR and annotated as the open reading frame 77 (ORF77), which belongs to protein families (Pfam) with accession PF10705 [110, 111]. In our study, *ycf15* gene is normally expressed in most *Justicia* plants, but pseudogenized in plastome of *J. adhatoda* due to its premature stop codons, which was doubted to be caused by gene degradation during RNA transcription [112, 113]. Meanwhile, this gene is different among other genera of Acanthaceae. For instance, it expresses under positive selection in *Dicliptera* [92], but acts as a pseudogene in *Echinacanthus* [91], or is even lost in *Barleria prionitis* L., *R. pectinata* and *S. cusia* [93, 98, 101]. Therefore, it is suggested that *ycf15* gene could be associated with plastome evolution of Acanthaceae, but its gene function remains to be further studied.

### IR structure variation

Chloroplast genome structure is highly conserved across angiosperms [66, 67]. This is especially true for the IR regions, which is caused by low substitution rates and strict copy correction during repeat sequences replication [114]. The IR often ranges in size from 7 to 88 kb in angiosperms [115–117], with the extent of IR due largely to expansions and contractions at the SSC and LSC boundaries [114]. In our study, *Justicia* is different from other genera of Acanthaceae in three IR borders, i.e., LSC/IRa, LSC/IRb and SSC/IRb (Fig. 3). Compared with *Justicia*, significant IR expansion from IR to LSC was found in *E. lofouensis* and *R. pectinata*, and IR contraction with two directions of boundary shifts from IR to LSC and SSC was also detected in *A. knappiae*, *C. nutans* and *S. cusia*. The discrepancy of IR borders of plastomes within *Justicia*, however, only performs on SSC/IRa and SSC/IRb regions of *J. grossa* and other *Justicia* species. In detail, the lengths of *ndhF* and *ycf1* in IRs are much shorter than those of other *Justicia* species, while the lengths of these two genes located in SSC are much longer than others, which is thought to undergo IR contraction and cause an increase length of SSC region. Therefore, it is indicated that *J. grossa* is different from other *Justicia* species at the genomic structure level.

According to the statistics of cp. genome structure types of Laminales [98], all the *Justicia* species belong to type II (a *rps19* pseudogene at the IR/LSC border). However, the plastome structure of *C. nutans*, with *ndhF* and *ycf1* boundary genes fully located in SSC, was not recorded before, thus it is firstly reported here. In our study, IR expansion and contraction events mainly contribute to genomic structure and sizes as well as gene composition variations among different genera of Acanthaceae, which is congruent with other plant lineages, including subfamily Commelinoideae (Commelinaceae Mirb.) [118], *Angelica* L. (Apiaceae Lindl.) [119], *Paphiopedilum* Pfitzer (Orchidaceae) [79] and *Balanites aegyptiaca* (L.) Delile (Zygophyllaceae R.Br.) [120]. It is suggested that IR expansion and contraction events will provide useful references for further research on plastid genome rearrangement of angiosperms, with an emphasis on gene content and evolution of the IRs.

### Potential molecular markers selection

Because the evolutionary rates of non-coding regions are faster than coding regions [76–78], LSC and SSC regions often exhibit higher sequence divergence than the IR regions in *Justicia* (Fig. 4), which is in accordance with other genera of Acanthaceae [91, 92, 94, 98]. Therefore, all of the mutational hotspots across the 13 *Justicia* complete cp. genomes were identified in single-copy regions (Fig. 5), of which six were intergenic spacer (*rps16-trnQ*, *rpoB-trnC*, *trnT-trnL*, *rps4-trnT*, *ccsA-ndhD* and *rps15-ycf1*), one was intron (*rpl16* intron) and one was protein coding gene (*ycf1*). The gene *ycf1* is a conservative homologous coding sequence with abundant variable sites [121–123]. Our phylogenetic topology based on *ycf1* gene is also generally identical with the cp. genome tree (Fig. S5). In addition, Dong et al. [121] also proposed that *ycf1* is the most promising plastid DNA barcode for land plants and plays an important role in genome evolution. Meanwhile, in some previous studies [123–125], *ycf1* gene has also been considered as an appreciated source to provide effective genetic information for phylogeny and species identification in breeding resources. Even one special concern for the use of *ycf1* as a barcode is the absence of *ycf1* in some taxa, such as Poaceae [121]. Therefore, this gene could be developed as a candidate DNA barcode for further phylogenetic reconstruction of *Justicia*. Compared with conserved coding regions, intergenic spacer and introns often show greater discrimination power at low taxonomic levels [126]. The *rpl16* intron and *trnT-trnL* have provided an effective molecular phylogeny in other plants, e.g., *Chusquea* Kunth (Poaceae) [127], *Echinochloa* P.Beauv. (Poaceae) and *Castanea* Mill. (Fagaceae Dumort.) [128, 129]. They were also proved to be a good resolution for phylogeny of Justicieae [9, 10]. Additionally, the five other non-coding regions have been proposed to be candidate DNA barcodes for phylogenetic research in other plant lineages, such as subfamily Dialioideae Azani et al. (Fabaceae Lindl.) [130], subfamily Zingiberoideae Hassk. (Zingiberaceae Martinov) [131], subfamily Allioideae Herb. (Amaryllidaceae J.St.-Hil.) [132], *Echinacanthus* (Acanthaceae) [91] and *Tetrastigma hemsleyanum* Diels & Gilg (Vitaceae Juss.) [133]. Therefore, it is believed that the eight mutational hotspots regions identified in our study could be potential molecular markers in *Justicia* phylogenetic studies. However, due to our results are only preliminary, more sampling and PCR amplification experiments for each primer of these barcodes should be carried out to test whether they could be feasible in phylogenetic research of *Justicia* in the future.

Simple sequence repeats, SSRs, known as microsatellites, are short stretches of DNA containing repetitive sequences of 1–6 bp in length, have been the most frequently used genetic marker in species identification and population genetics [134], due to their co-dominant inheritance and high polymorphism [135]. SSRs are the same units with different repeat numbers located in the homologous regions and these regions are frequently used to identify variable species [92, 117, 136–138]. Therefore, cp. SSRs were identified in our study. As a result, repetitive sequences are significantly variable among different species (Figs. 6 and 7). Most cp. SSRs are located in intergenic spacer of LSC and SSC regions, with 61% in non-coding regions and only a small amount in protein-coding genes (25%) and introns (14%) (Table S6), which is consistent with other plants [91, 92, 139]. It is revealed that non-coding regions are more variable to screen valuable polymorphic SSRs [140–142]. Besides, cp. SSRs that are polymorphic within and among species can provide unique insights into species identification and their purities, particularly on those economically important plants [140]. Thus, a total of 91 polymorphic SSRs were identified here (Table 3). Due to the high similarity of universal DNA barcodes (*matK*, *rbcL*) among *Justicia* species (Table S4), our selected polymorphic SSRs can be effective genetic markers to identify these species. As the most common repeat unit, mononucleotide is mainly located in intergenic spacer and attributed to almost 90% A/T base richness (Table S6), which is in line with other plants [87, 141, 143]. Notably, apart from the highly variable hotspots region as mentioned above, *ycf1* is also detected as the most polymorphic gene with five different motifs (AATT, TTTC, TTA, TCT and T) in the 13 *Justicia* species (Table 3). Based on our results, it is believed that this gene is the most promising molecular marker for species identification in *Justicia* in the future. Importantly, based on our results of repetitive sequences analyses, it is indicated that *J. grossa* is quite different from other *Justicia* species owing to its richest SSRs and dispersed repeats among all the *Justicia* species, with an emphasis on the number of mononucleotide and dispersed repeats of over 50 bp (Figs. 6 and 7).

### Potential reason for the low support values of ITS tree

The tree topology based on ITS sequence is generally similar with those based on whole chloroplast genome and 77 common cpCDS datasets, but the ITS tree has low support values whether based on ML or Bayes algorithm (BS < 70, PP < 0.95) (Fig. 8, Fig. S1–S4). In this case, the low support values are mainly attributed to the insufficiency of variable sites, though evolutionary rates of nuclear are faster than plastid. In our results, the alignment of plastid genomes has much more variable sites in total (56,714) than ITS (457) (see Results part). Therefore, our ITS tree caused the sampling error, which means that in the process of substitution model selection, explaining too many parameters with too little data increases variance of estimable models [144, 145]. Anyway, phylogenetic analyses of too short sequences are more prone to result in sampling error than long sequences, simply because they contain less phylogenetic information [146].

### Phylogenetic relationships of Asian ***Justicia*** plants

Recently, in the most comprehensive work of Graham [1], *Justicia* was divided into nine sections and seven subsections based on the combination of morphological characters of inflorescence, stamen, pollen, fruit and seed traits. In our study, we sampled 13 *Justicia* species from seven Asian *Justicia* sections. The phylogenetic results based on whole plastome, both 77 common protein-coding genes and ITS datasets (Fig. 8, Fig. S1–S4) suggest that *Justicia* s.l. is a polyphyletic group, which is supported in previous studies based on several molecular markers [8–12, 147].

In our results, Justicieae can be divided into three clades, i.e., Clade I, II and III (Fig. 8). Clade I contains two species, i.e., *J. grossa* and *C. nutans*, and might be assigned to subtribe Tetramerinae. *J. grossa* is isolated with other species in *Justicia* and forms the sister group with *C. nutans*. This result is also in accordance with previous phylogenetic studies using several molecular markers [9, 11]. *J. grossa* belongs to sect. *Grossa* B. Hansen. Sect. *Grossa* comprises three species from China, Vietnam, Laos, Thailand, Malaysia and Myanmar, and, morphologically, it is quite different from other *Justicia* plants in its bithecous anther having a solid, cusp-like spur at the base of each theca (Fig. S6), but other *Justicia* species only spurred on the lower theca [6, 8, 9, 11, 148]. Meanwhile, sect. *grossa* is also different from *Clinacanthus* in its bithecous anthers with both spurred thecae while *Clinacanthus* has muticous monothecous anthers [3, 6]. Therefore, *J. grossa* may be recognized as a new undescribed genus. However, the further phylogenetic research is necessary to determine the position of sect. *Grossa* since only one species was sampled in our study.

With the exception of *J. grossa*, all of the remaining Asian *Justicia* species may be assigned to subtribe Justiciinae and can be divided into two main clades, i.e., Clade II and III. Clade II contains *J. latiflora*, *J. lianshanica*, *J. leptostachya*, *J. patentiflora* and *J. gendarussa* together with *Rungia*. Clade III includes *J. adhatoda*, *J. betonica*, *J. demissa*, *J. mollissima*, *J. procumbens*, *J. quadrifaria* and *J. vagabunda* together with *Dicliptera*. In terms of morphology, those plants of Clade II have the fruits in which the placenta separated from the capsule wall but remain attached at the apices causing them to rise up at dehiscence while the fruits not as such in Clade III [1, 9].

**Fig. 9:**
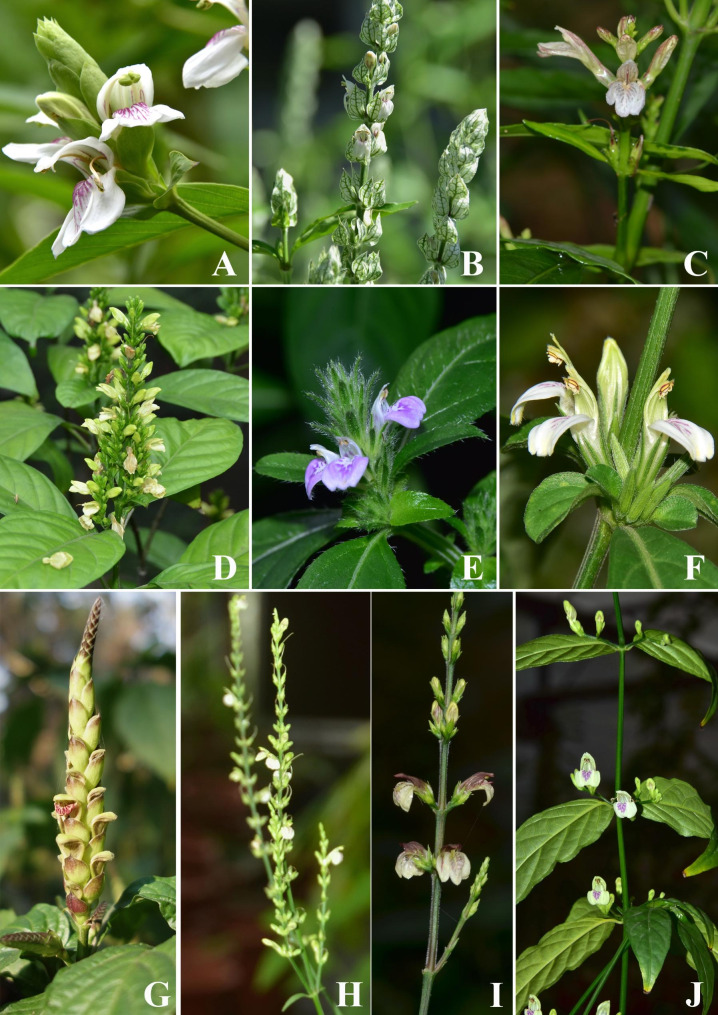
Morphological characters of ten representative Asian *Justicia* plants. (**A**). *J. adhatoda* L.; (**B**). *J. betonica* L.; (**C**). *J. gendarussa* N. J. Burman; (**D**). *J. grossa* C. B. Clarke; (**E**). *J. procumbens* L.; (**F**). *J. quadrifaria* (Nees) T. Anderson; (**G**). *J. latiflora* Hemsl.; (**H**). *J. leptostachya* Heml.; (**I**). *J. lianshanica* (H. S. Lo) H. S. Lo; (**J**). *J. vagabunda* Benoist

In Clade II, *J. gendarussa* is clustered with *J. ventricosa* and closely related to *Rungia* in terminal spike and elastic placenta when fruit dehiscence [3, 9]. Four species (*J. latiflora*, *J. lianshanica*, *J. leptostachya* and *J. patentiflora*) are clustered together in sharing the characters of elongated simple or rarely branched terminal spikes, narrow bracts subtending the small flowers or clusters of small flowers (Fig. 9), as well as 2-colporate pollen grains and rugulose seeds [26, 149, 150].

In Clade III, *J. adhatoda* and *J. betonica* were considered to be closely related by Graham [1], but differs from each other mainly in the flower number at each node of the spike and the bract shape [1, 30]. The former has the spikes with one flower per node and ovate-oblong bracts while the latter has the spikes with two flowers per node and white cordate bracts with green veins (Fig. 9). The next diverging species is *J. quadrifaria*, which is distributed in Asia and Africa and is characterized by the axillary cymose inflorescence, tiny subulate or triangular bracts and 5-partite calyx with equal segment (Fig. 9) [3, 5, 7, 29]. Next to diverge is the group including three species *J. demissa*, *J. mollissima* and *J. procumbens*, sharing the characters of short simple terminal spikes and 5-partite calyx with one extremely reduced segment and purplish red corolla (Fig. 9) [1, 3, 5]. In our analysis, *J. vagabunda* is the last diverging species and is sister to *Dicliptera*, but distantly related to other members of *Justicia* (Fig. 8). It differs from other sampled *Justicia* species in having axillary cymes and irregularly rounded-rugose tuberculate seeds. Besides, it is also easily distinguished from *Dicliptera* by its lower anther-theca spurred at base and the placenta not separate from the capsule wall while the anther and fruit not as such in the latter [3, 6, 151].

Interestingly, we discovered that the position and rachis internode of inflorescence of *Justicia* plants vary from terminal to axillary, spikes gradually shorten into cymes, seems to be a phenotype positively correlated with its evolutionary history. However, to fully resolve phylogenetic relationships of *Justicia*, more genetic resources and morphological evidence of Justicieae species from Africa, Australia and South America need to be combined with our Asian taxa for phylogenetic analyses in the future.

## Conclusions

Our study sequenced 12 complete chloroplast genomes of Asian *Justicia* plants and combined with the previously published plastome of *J. leptostachya* for further comparative genomic analyses. The 13 *Justicia* cp. genomes are highly conserved in genome structures, organizations and gene contents. However, the gene *ycf15* was found to be a pseudogene in *J. adhatoda* while normally expressed in others. Four IR/SC junctions of plastomes are generally identical within genus with the exception of *J. grossa*. Repetitive sequences are significantly variable at the interspecific level. A total of 91 polymorphic SSRs and the eight mutational hotspots were also identified. Among them, the gene *ycf1* is the most promising plastid DNA barcode for *Justicia* species identification and phylogenetic studies. Our phylogenetic results strongly supported that *Justicia* is polyphyletic and shed lights on the relationships among Asian *Justicia* plants for the first time. Interestingly, the evolutionary history of *Justicia* coincides with morphology of inflorescence position from terminal to axillary and spikes gradually shorten into cymes.

Additionally, it is noteworthy that *J. grossa* is different from other *Justicia* species in the following three aspects: (i) it is richest in SSRs and dispersed repeats compared with other *Justicia* species; (ii) its SC/IR boundary genes *ndhF* and *ycf1* located in IRs are much shorter than other *Justicia* species, while*Ψycf1* located in SSC is much longer than others; (iii) its systematic position is in subtribe Tetramerinae, which is distantly related to other members of *Justicia*. Therefore, combined with its morphology of bithecous anthers with both spurred thecae, *J. grossa* should be defined as a new genus. Our study may not only improve the understanding of plastomes of *Justicia* plants, but also provide more genetic information for further researches on the evolutionary history of *Justicia* in the future.

## Materials and methods

### Sampling, DNA extraction and sequencing

A total of 12 plants from seven Asian sections of *Justicia* were sampled in our study, followed by classification system of Graham [1] and Hansen [148], including *J. adhatoda*, *J. betonica*, *J. demissa*, *J. gendarussa*, *J. grossa*, *J. latiflora*, *J. lianshanica*, *J. mollissima*, *J. quadrifaria*, *J. patentiflora*, *J. procumbens* and *J. vagabunda*. Fresh and healthy leaves of these 12 *Justicia* plants were collected in the field, with sampling information listed in Table S1. Leaf samples were immediately dried with silica gel for further DNA extraction. All the voucher specimens were deposited in the Herbarium of South China Botanical Garden, Chinese Academy of Sciences (IBSC).

Total genomic DNA was isolated using the modified CTAB method [152]. The extracted genomic DNA was sent to the Beijing Genomics Institute (BGI) for qualification control by fluorometer (QubitFluorometer, Invitrogen). DNA samples of concentration up to standard (≥ 1 μg) were randomly sheared into fragments by Covaris M220 (Covaris, Woburn, MA). Insert size of 270 bp DNA fragments were enriched by PCR, and the paired-end (2 × 150 bp) libraries were constructed on the Illumina HiSeq 4000 platform. Finally, about 2 Gb genome skimming data were generated.

### Assembly and annotation of whole plastome and nrDNA

To improve assembly accuracy and efficiency, Trimmomatic v 0.39 was first employed to filter out unpaired and low-depth reads from clean data using default parameters [153]. The filtered clean reads were utilized to *de novo* assemble complete cp. genomes using GetOrganelle v 1.6.2 pipeline [154]. To obtain complete cp. genomes and nrDNA sequence, six k-mer values, including 21, 45, 65, 85, 105,125, were set for plastid contigs connection. Subsequently, the filtered plastid reads were transferred to Bandage [155] software for visualization processing. Two opposite plastid sequences exported from Bandage were aligned with the reference sequence *Andrographis paniculata* (GenBank accession no. KF150644), and one that matched the reference was screened on the annotation of PGA software [156] and the Annotation of Organellar Genomes (GeSeq) [157]. The final annotations of plastomes and nrDNA sequences were manually corrected in Geneious Prime v 9.1.4 [158]. The whole cp. genomes and nrDNA maps were drawn by using OGDRAW v 1.3.1 (https://chlorobox.mpimp-golm.mpg.de/) with default settings [159].

### Genome divergences comparison and codon usage analyses

The complete cp. genome of *J. leptostachya* was combined with newly assembled 12 cp. genomes in our study for further comparative genomic analyses. Whole plastomes of the 13 *Justicia* species and seven species of other genera in Acanthaceae were combined to visualize IR expansion and contraction by using IRscope online software (https://irscope.shinyapps.io/irapp/) [160]. Besides, the 13 *Justicia* plastomes were aligned and globally viewed using the online mVISTA program [161] (https://genome.lbl.gov/vista/index.shtml) in Shuffle-LAGAN mode [162], with the annotation of *J. leptostachya* as the reference. To evaluate nucleotide diversity (Pi), MAFFT v 7.450 [163] was operated to align the 13 *Justicia* cp. genomes. Then, Pi value was implemented based on a sliding window by Dnasp v 5.0 [164], with step size of 200 bp and window length of 800 bp. Relative synonymous codon usage (RSCU) in all the protein-coding sequences of 13 *Justicia* plants were calculated using CodonW v 1.4.2 software with default parameters [165].

### Repetitive sequences analyses

Dispersed repeats among the 13 *Justicia* cp. genomes were identified with four directions (forward, reverse, palindromic, and complement) using the online REPuter program (https://bibiserv.cebitec.uni-bielefeld.de/reputer) [166], with the maximum computed repeats number of 100 and the minimal repeat size of 20 bp. The program MISA [167] was employed to obtain multiple short tandem repeats, including mononucleotide (mono-), dinucleotide (di-), trinucleotide (tri-), tetranucleotide (tetra-), pentanucleotide (penta-), and hexanucleotide (hexa-) SSRs, with corresponding minimum repeat units set as 10, 6, 3, 3, 3, 3. Tandem repeats were also identified using Tandem Repeats Finder v 4.09 [168].

### Phylogenetic analysis

Three datasets containing whole chloroplast genome (WCG), plastid protein-coding genes (PCG) and internal transcribed spacer (ITS) were designed for phylogenetic analysis based on two different algorithms including Maximum Likelihood (ML) and Bayesian Inference (BI). For WCG tree, a total of 62 samples were utilized for phylogenetic tree reconstruction, comprising 12 newly sequenced *Justicia* cp. genomes in our study, three previously published *Justicia* cp. genomes and 46 cp. genomes of other genera belonging to Acanthaceae from GenBank. *Sesamum indicum* L. (JN637766) was selected as the outgroup species because it belongs to the family Pedaliaceae R.Br., which is most closely related to Acanthaceae based on APG IV (https://www.mobot.org/MOBOT/research/APweb/). For PCG tree, with the exclusion of *psbA*, *rpl2* and *ycf15* gene due to lacking in some genera, a total of 77 common protein-coding genes were extracted from whole plastomes by using a python script ‘get_annotated_regions_from_gb.py’ (https://github.com/Kinggerm/PersonalUtilities/). Gblocks v 0.91b [169] was further employed to trim each gene matrix. The parameters are set as allowing up to half of the samples to have missing data and at least 87 minimum sequence length per gene matrix. For ITS tree, a total of 63 samples were utilized for phylogenetic inference, including 13 *Justicia* ITS sequences extracted from our nrDNA data by Geneious Prime and 50 previously published ITS sequences of Acanthaceae from GenBank. *Strobilanthes cusia* (Nees) Kuntze was set as the outgroup for the ITS tree. All the GenBank accession numbers of cp. genomes and ITS sequences used for our phylogenetic analyses were listed in Table S2.

Then, the three datasets were aligned by using MAFFT and the test for nucleotide substitution saturations was implemented in DAMBE v 7.2.133 referring to Xia’s method [170], with a significance threshold of Iss < Iss.c and p-value < 0.05. ML analyses were conducted by RAxML v 8.0.0 [171], with the best-fit parameter settings as rapid bootstrap algorithm and GTRGAMMAI model recommended by jModelTest v 2.1.6 [172]. The number of 12,345 was specified as the random seed of parsimony tree inference with 1000 replicates performed. BI analyses were operated by using MrBayes v3.2.2 [173], with the best-fit model selected as SYM + G inferred from MrModeltest v 2.3 [174]. Rates of variations across sites were trimmed as gamma. For each analysis, two simultaneous runs of four Monte Carlo Markov Chains (three heated and one cold) were run for six million generations with a random tree as the starting point and saving trees every 1000 generations. After rejecting the first 25% burn-in samples, the optimized topology with posterior probabilities (PP) > 0.95 was generated. Finally, the phylogenetic results were visualized with FigTree v 1.4.3 (http://tree.bio.ed.ac.uk/software/figtree/).

### Electronic supplementary material

Below is the link to the electronic supplementary material.

**Additional file 1: Table S1**. Collection and assembly information of 12 *Justicia* species

**Additional file 2: Table S2**. All the GenBank accession numbers used for phylogenetic analyses utilized in our study

**Additional file 3: Table S3**. Gene sizes of all the protein-coding genes of 13 *Justicia* chloroplast genomes

**Additional file 4: Table S4**. Genes with introns in the 13 *Justicia* chloroplast genomes, including the exon and intron lengths

**Additional file 5: Table S5**. Codon usage bias of 20 amino acids within 13 *Justicia* chloroplast genomes

**Additional file 6: Table S6**. Quantity statistics of SSRs of the 13 *Justicia* chloroplast genomes

**Additional file 7: Figure S1**. ML phylogram for 62 taxa of Acanthaceae based on 77 common protein-coding genes

**Additional file 8: Figure S2**. BI phylogram for 62 taxa of Acanthaceae based on 77 common protein-coding genes

**Additional file 9: Figure S3**. ML phylogram for 63 taxa of Acanthaceae based on ITS sequence

**Additional file 10: Figure S4**. BI phylogram for 63 taxa of Acanthaceae based on ITS sequence

**Additional file 11: Figure S5**. Phylogenetic reconstruction for *Justicia* species and other genera of Acanthaceae based on *ycf1* gene

**Additional file 12: Figure S6**. Morphology of inflorescence and anther of *Justicia grossa*

## Data Availability

All the voucher specimens were deposited in the Herbarium of South China Botanical Garden, Chinese Academy of Sciences (IBSC), and their sampling information is listed in Table S1. All the newly sequenced 12 cp. genomes in this study are available in National Center for Biotechnology Information (NCBI) (https://www.ncbi.nlm.nih.gov), with accession numbers: MN848243–MN848252 and MN885664–MN885665 (Table 1). All the newly sequenced 13 nrDNAs in this study are available in NCBI with accession numbers: OQ785888–OQ785900. All the GenBank accession numbers of previously published sequences used for phylogenetic analyses in our study can be found in Table S2.

## Abbreviations

BI: Bayesian inference
BS: Bootstrap
CDS: Coding regions
CNS: Non-coding regions
cp: Chloroplast
HIV: Human immunodeficiency virus
IR: Inverted repeat
ITS: Internal transcribed spacer
LSC: Large single-copy region
ML: Maximum likelihood
nrDNA: Nuclear ribosome DNA
NW: New Word
ORF77: Open reading frame 77
OW: Old Word
PCG: Plastid protein-coding genes
Pfam: Protein families
Pi: Nucleotide diversity
PP: Posterior probability
RSCU: Relative synonymous codon usage values
SD: Shine-Dalgarno
SSC: Small single-copy region
SSRs: Simple sequence repeats
UTR: Untranslated regions
WCG: Whole chloroplast genome
