# Supplementary figures and images for: Complete plastid genome structure of 13 Asian Justicia (Acanthaceae) species: comparative genomics and phylogenetic analyses
Source: BMC Plant Biol. 2023 Nov 15;23:564. doi: 10.1186/s12870-023-04532-0 (PMC10647099; doi:10.1186/s12870-023-04532-0)

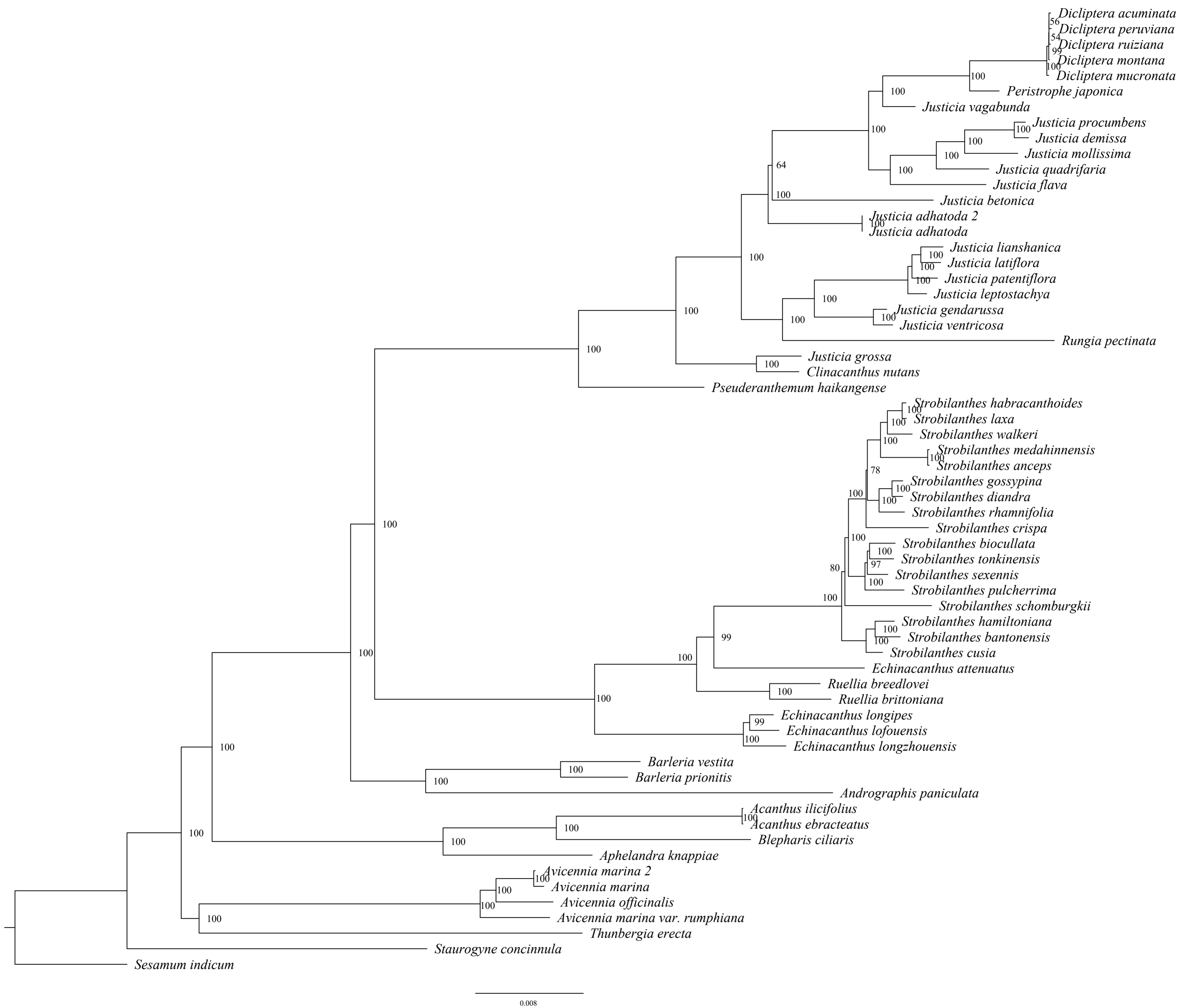

**Fig. S1** ML phylogram for 62 taxa of Acanthaceae based on 77 common protein-coding genes.

Supplement: Supplementary file 7 — Additional file 7: Figure S1. ML phylogram for 62 taxa of Acanthaceae based on 77 common protein-coding genes [file 12870_2023_4532_MOESM7_ESM.pdf]

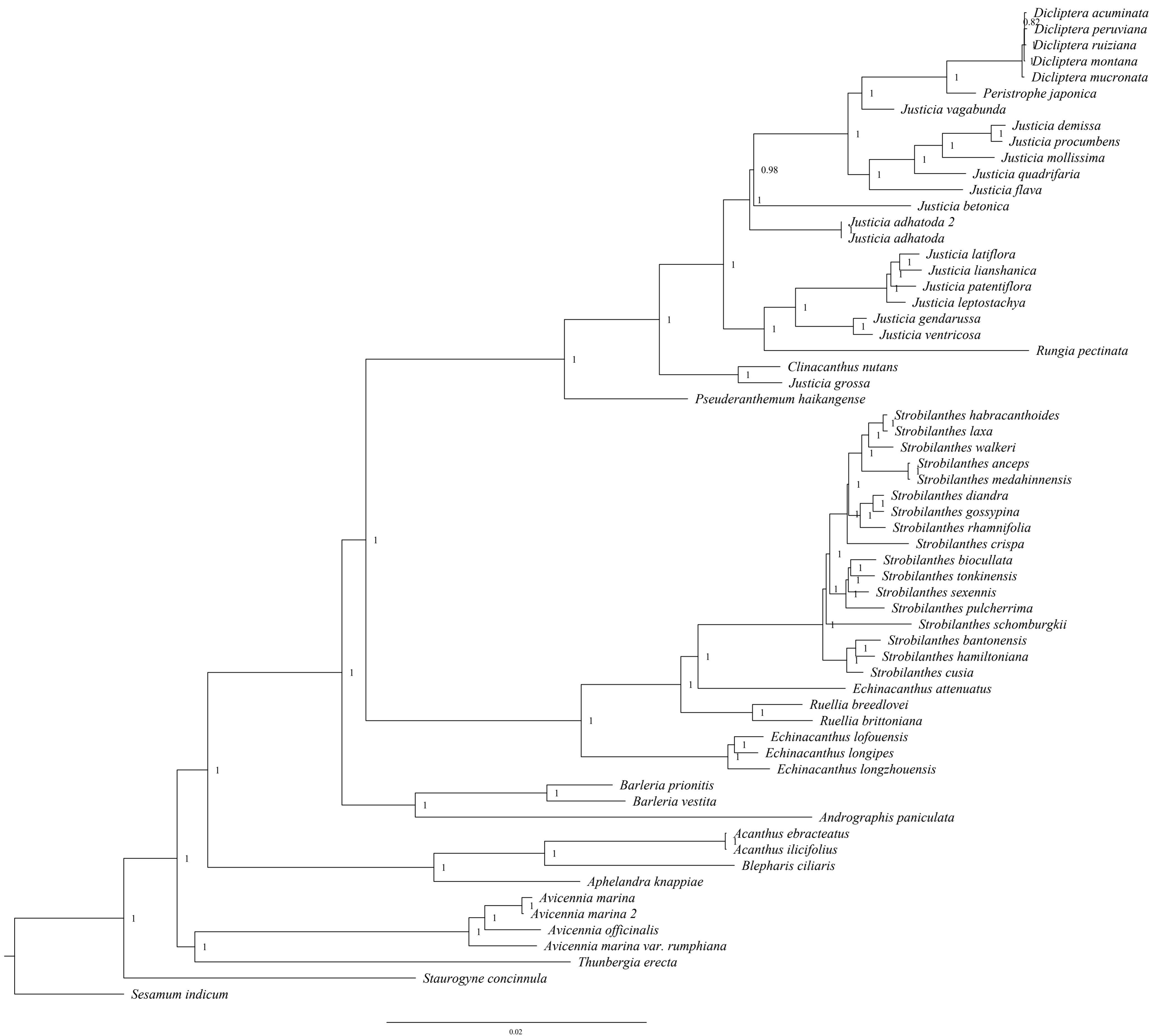

**Fig. S2** BI phylogram for 62 taxa of Acanthaceae based on 77 common protein-coding genes.

Supplement: Supplementary file 8 — Additional file 8: Figure S2. BI phylogram for 62 taxa of Acanthaceae based on 77 common protein-coding genes [file 12870_2023_4532_MOESM8_ESM.pdf]

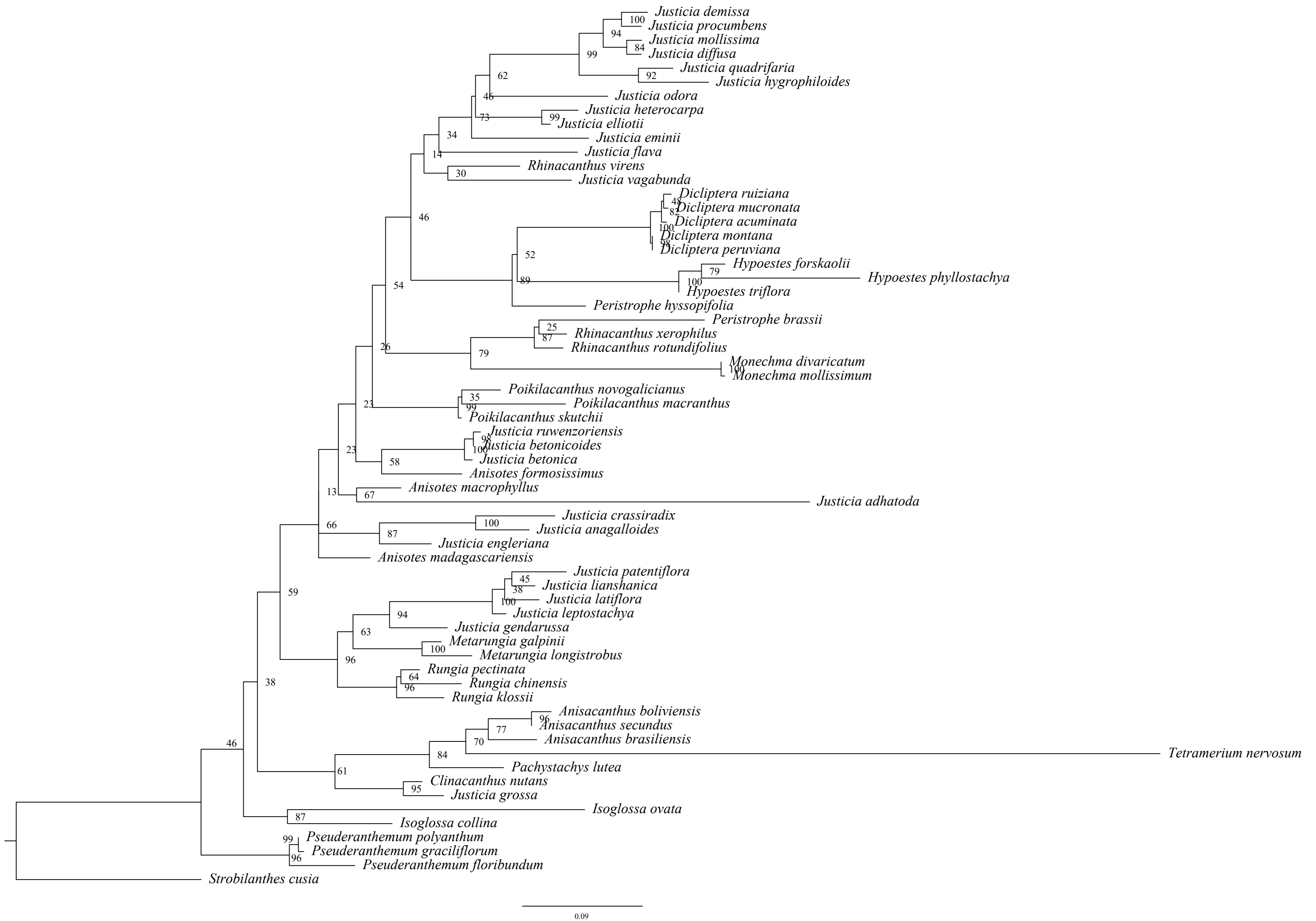

**Fig. S3** ML phylogram for 63 taxa of Acanthaceae based on ITS sequence.

Supplement: Supplementary file 9 — Additional file 9: Figure S3. ML phylogram for 63 taxa of Acanthaceae based on ITS sequence [file 12870_2023_4532_MOESM9_ESM.pdf]

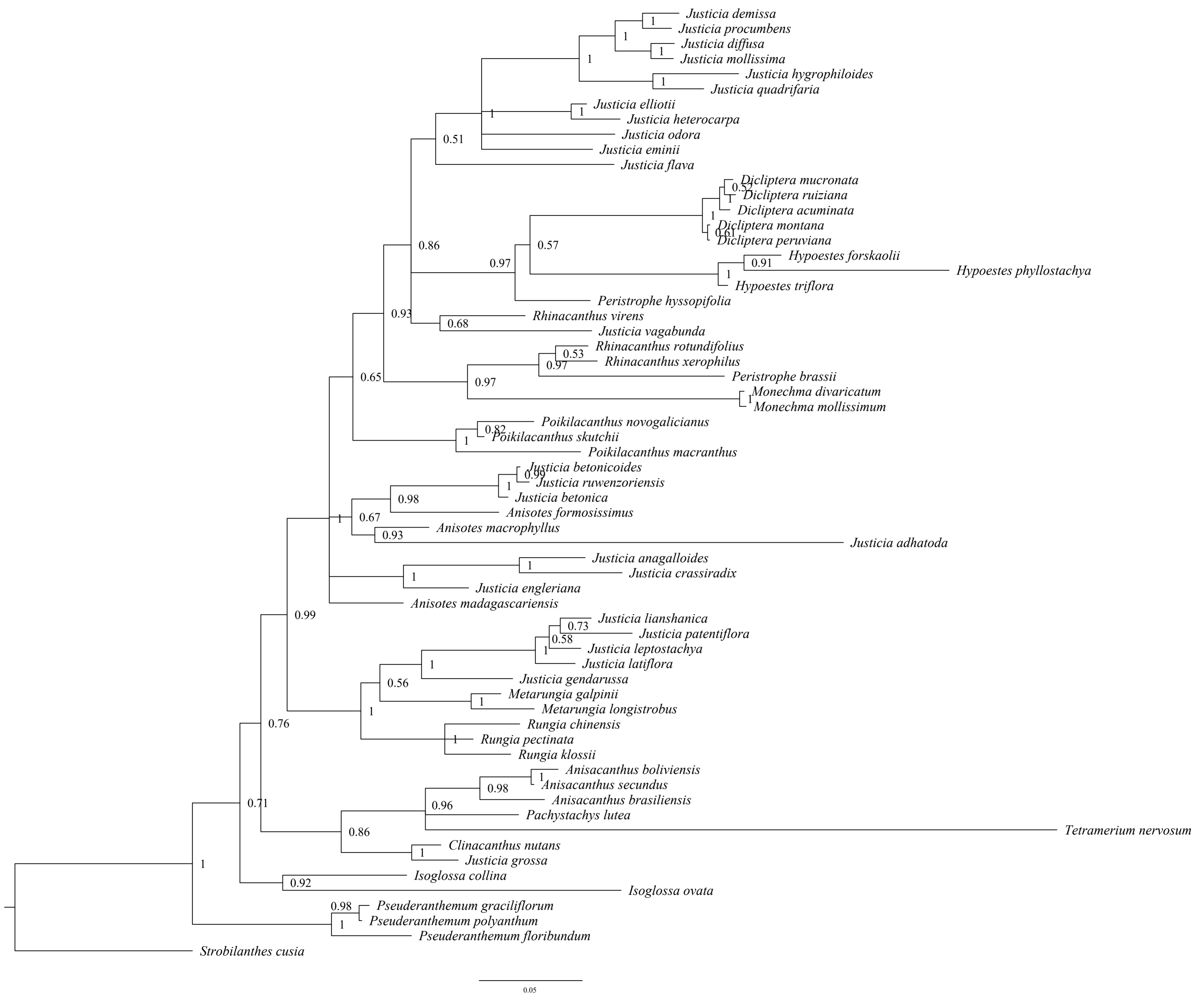

**Fig. S4** BI phylogram for 63 taxa of Acanthaceae based on ITS sequence.

Supplement: Supplementary file 10 — Additional file 10: Figure S4. BI phylogram for 63 taxa of Acanthaceae based on ITS sequence [file 12870_2023_4532_MOESM10_ESM.pdf]

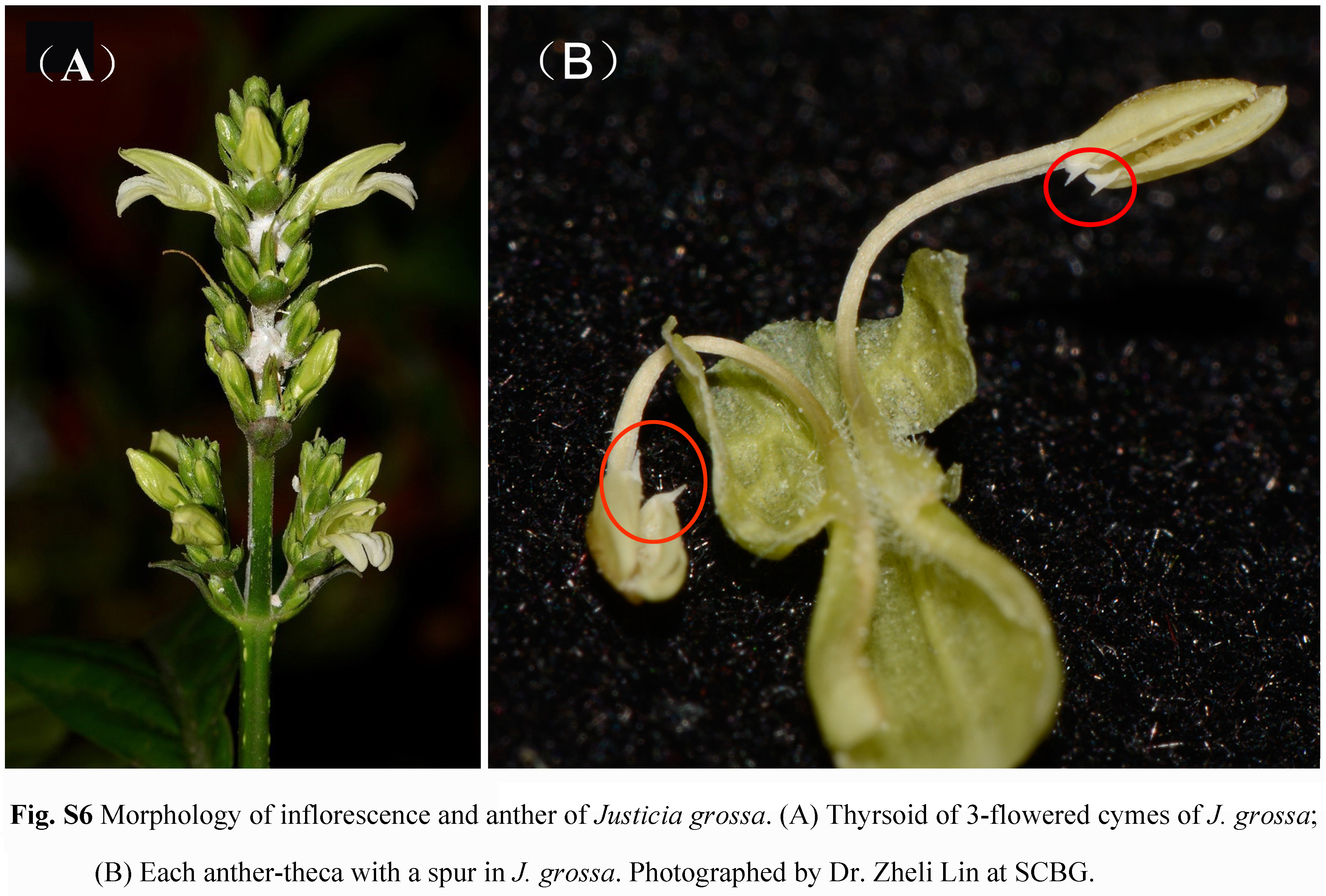

Supplement: Supplementary file 12 — Additional file 12: Figure S6. Morphology of inflorescence and anther of Justicia grossa [file 12870_2023_4532_MOESM12_ESM.jpg]
