# Supplementary material for: Complete plastid genome structure of 13 Asian Justicia (Acanthaceae) species: comparative genomics and phylogenetic analyses
Source: BMC Plant Biol. 2023 Nov 15;23:564. doi: 10.1186/s12870-023-04532-0 (PMC10647099; doi:10.1186/s12870-023-04532-0)
Supplement: Supplementary file 11 — Additional file 11: Figure S5. Phylogenetic reconstruction for Justicia species and other genera of Acanthaceae based on ycf1 gene [file 12870_2023_4532_MOESM11_ESM.pdf]

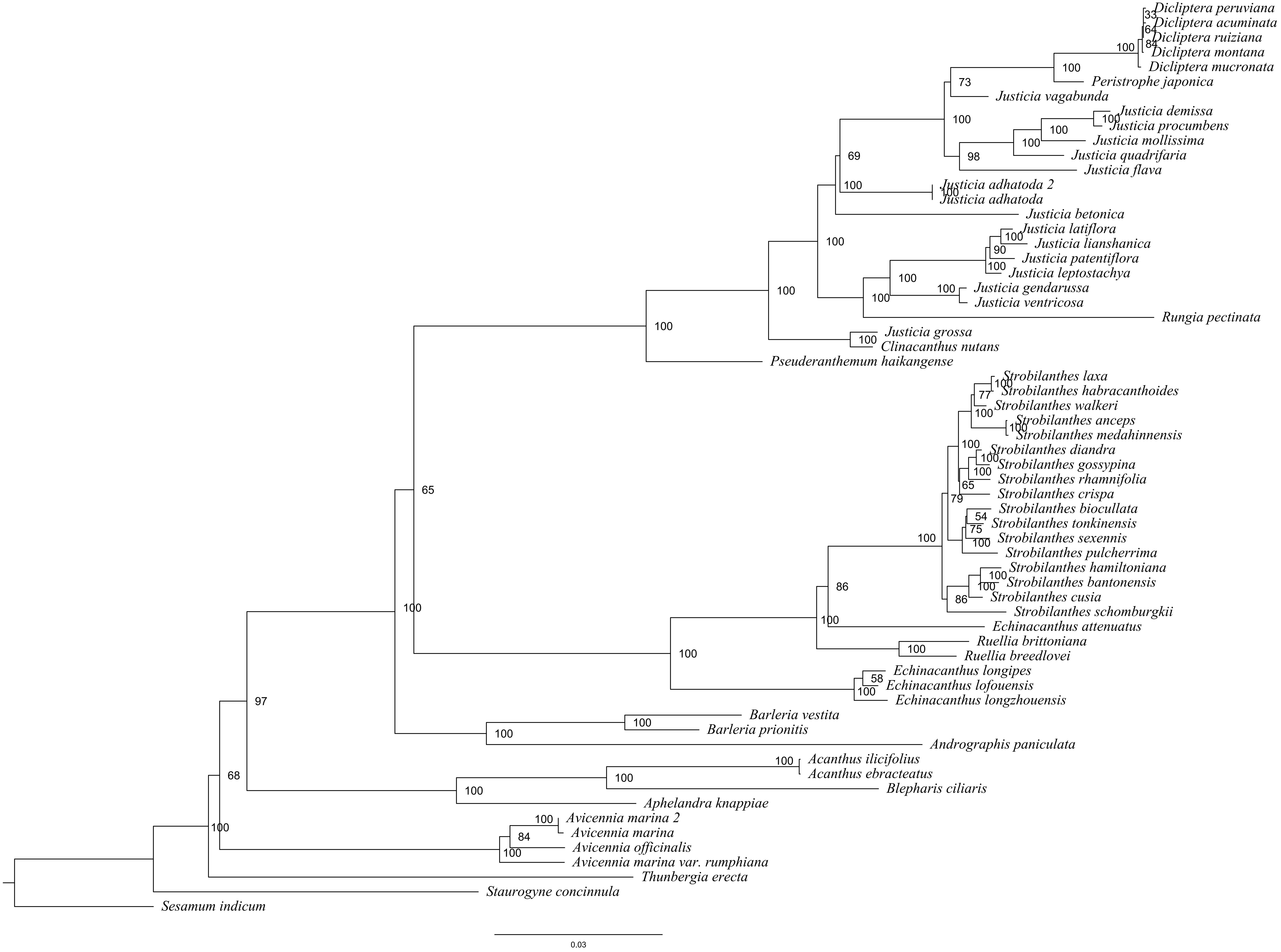

Fig. S5 Phylogenetic reconstruction for *Justicia* species and other genera of Acanthaceae based on *ycf1* gene.
